# Cold suppresses virus accumulation and alters the host transcriptomic response in the turnip mosaic virus–*Arabidopsis halleri* system

**DOI:** 10.1093/pcp/pcaf010

**Published:** 2025-01-18

**Authors:** Mie N Honjo, Naoko Emura, Mari Kamitani, Hiroshi Kudoh

**Affiliations:** Center for Ecological Research, Kyoto University, Hirano 2-509-3, Otsu 520-2113, Japan; Center for Ecological Research, Kyoto University, Hirano 2-509-3, Otsu 520-2113, Japan; Faculty of Agriculture, Kagoshima University, Korimoto 1-21-24, Kagoshima 890-0065, Japan; Center for Ecological Research, Kyoto University, Hirano 2-509-3, Otsu 520-2113, Japan; CiRA Foundation, Kyoto University, Shogoin kawahara-cho 53, Sakyo-ku, Kyoto 606-8397, Japan; Center for Ecological Research, Kyoto University, Hirano 2-509-3, Otsu 520-2113, Japan

**Keywords:** RNA-seq, temperature dependence, turnip mosaic virus, virus inoculation

## Abstract

Since plant viruses cause lifelong infections, virus–plant interactions are exposed to large temperature fluctuations in evergreen perennials. In such circumstances, virus–plant interactions are expected to change significantly between the warm and cold seasons. However, few studies have investigated the effects of cold temperatures on virus–plant interactions. Here, we show that in a persistent infection system of the turnip mosaic virus (TuMV)–*Arabidopsis halleri*, cold temperatures lead to slow viral replication/spreading within the host, attenuated host symptoms, and cold-specific transcriptomic responses. Many differentially expressed genes (DEGs) were detected between virus-inoculated and mock-inoculated plants under warm and cold conditions; however, the sets of DEGs and response timings were temperature-dependent. At cold temperatures, the expression of photosynthesis-related genes decreased in the early stages of infection. However, it recovered to the same level as that in uninfected plants in the later stages. In contrast, the transcriptomic changes under warm conditions suggest that viral infections cause auxin signaling disruption. These responses coincided with the inhibition of host growth. We identified 6 cold- and 38 warm-specific DEGs, which changed their expression in response to TuMV infection under more than half of the conditions for either cold or warm temperatures. Further validation of the putative relationships between transcriptomic and phenotypic responses of the host is required. Our findings on temperature-dependent host responses at both symptomatic and transcriptomic levels help us understand how warm and cold temperatures affect virus–plant interactions in seasonal environments.

## Introduction

A recent understanding of plant viruses in natural systems is that they can widely infect wild plants for long periods without causing significant damage to their hosts (Roossinck 2012, Honjo et al. 2020). One of the critical determinants of host–virus relationships is the combination of host and virus species/strains, and the surrounding environment is another crucial determinant (Hull 2014). Temperature is one of the most influential environmental factors that modify plant virus–host interactions by affecting virus accumulation and host responses (Harrison 1956). In temperate and boreal regions, where temperature regimes vary significantly between summer and winter, natural plant virus–host systems experience cold and warm temperatures. Although the temperature dependence of virus–plant interactions has been studied extensively, knowledge of alteration of these interactions under winter temperature regimes, 10°C and lower, remains limited.

The accumulation of viruses within an individual host plant is achieved by several processes, including viral replication at the initial site of infection, cell-to-cell movement via plasmodesmata, systemic spread through the vascular system, and propagation at the spread site (McLean et al. 1993). Each of these processes is affected by temperature. Previous studies on the temperature dependence of virus accumulation have found that the optimal temperature is specific to the virus–host combinations and often in the range of 20°C–28°C. These studies also reported that virus accumulation was suppressed when the temperature was 5°C–10°C lower than the optimal temperature. For example, the optimal temperature for virus accumulation of turnip mosaic virus (TuMV) in Chinese cabbage, *Brassica campestris*, was 23°C–28°C, and virus accumulation was highly suppressed at 13°C (Chung et al. 2015). For tobacco mosaic virus (TMV) in tobacco, *Nicotiana tabacum*, the optimal temperature was 24°C, but virus accumulation was much slower at 16°C (Lebeurier and Hirth 1966). The optimal temperature for virus accumulation was 28°C for cucumber mosaic virus (CMV) infected with tobacco leaves, and the level of virus accumulation was less than half at 18°C, 6 days after the infection (Zhao et al. 2016). A lower optimal temperature of 17°C has been reported for soil-borne wheat mosaic virus (SBWMV) infected with barley *Hordeum vulgare*, known to overwinter in its host (Ohsato et al. 2003). However, RNA replication of SBWMV was significantly reduced at 15°C compared to 17°C (Ohsato et al. 2003). These examples suggest that winter temperatures would suppress virus accumulation; however, viral accumulation at 10°C or below has rarely been studied.

Host responses to viral infection occur at multiple levels, including chloroplast and cell deformation, leaf symptoms, and whole-plant growth, and these responses are often temperature-dependent (Hull 2014). In response to viral infections, plant immunity, including salicylic acid (SA) signaling and RNA-silencing pathways, is usually induced to prevent or slow the viral spread. Temperature dependence of antiviral defense has been reported in some cases (Szittya et al. 2003). For example, a potato cultivar developed visible systemic symptoms at 30°C but not at 22°C after inoculation with potato virus Y (Nie et al. 2015). With TuMV in Chinese cabbage, symptoms developed 6 days after infection at 23°C–28°C, but no symptoms appeared within 30 days at 13°C (Chung et al. 2015). These examples suggest that low temperatures affect multiple aspects of virus–plant interactions, allowing the host to remain healthy.

To capture the complex nature of virus accumulation and host responses, transcriptome analyses using microarrays and RNA-seq have been applied in recent studies of virus–plant interactions (Whitham et al. 2003, Li et al. 2018, Liu et al. 2019). Differentially expressed genes (DEGs) between virus-inoculated and control plants often include defense genes involved in SA signaling and RNA-silencing, stress-responsive genes, heat shock proteins (HSPs), hormone-related genes, and photosynthesis-related genes (Whitham et al. 2006). Responses vary between inoculated and systemic tissues and change dynamically along the time course after the virus inoculation (Obrępalska-Stęplowska et al. 2015). Although how temperature interacts with viral infection in determining the host transcriptome is still largely unknown, RNA-seq analyses will provide comprehensive information on the response of host plants to viral infection under winter temperature regimes. They will allow the understanding of different responses under the optimal and winter temperature conditions.

The presence of warm and cold temperatures in a year may contribute to the persistence of virus–plant systems by allowing virus propagation during warm periods and enhancing host vigor during cold periods. We have previously reported that winter is particularly important for the persistence of TuMV in its natural host, *Arabidopsis halleri* subsp. *gemmifera* (*A. halleri*, hereafter) (Honjo et al. 2020). Because virus accumulation in new leaves is suppressed in winter (Honjo et al. 2020), we speculated that virus suppression by cold winters allows the host to survive. However, it is unclear how cold temperatures alter virus–host interactions and the effects of viruses on host symptoms and growth. Further experimental studies are required to compare the effects of cold and warm temperatures on viral accumulation, host growth, and transcriptomic responses.

In this study, we conducted temperature-manipulation experiments using a TuMV–*A. halleri* system derived from a natural habitat. Warm (25°C/20°C, day/night) and cold (10°C/5°C, day/night) conditions were used. The former is close to the optimal temperature for TuMV accumulation and symptom development in *Brassica* crops (Chung et al. 2015). The latter is the temperature regime experienced by the natural populations of *A. halleri* in winter and early spring (Honjo et al. 2020). We examined viral accumulation and host transcriptomic responses at 8, 15, and 28 days postinoculation (dpi). We designed our experiment for a more extended period (i.e. 28 dpi) based on the expectation that TuMV would take time to accumulate under cold conditions. In addition, we included 28 dpi to examine the long-term effects of viral infection in addition to short-term transient responses. We predicted that only some specific genes respond to viral infection, such as those involved in antiviral defense, plant hormone–related genes involved in growth response, and genes involved in the maintenance of the photosynthetic apparatus associated with disease symptoms.

Two questions were addressed in this study: (i) what are the patterns of intraplant virus accumulation and plant growth following viral inoculation under warm and cold conditions? (ii) How does the transcriptomic response to viral infection differ between cold and warm conditions?

## Results

### TuMV accumulation, host growth, and symptoms in the warm and cold

**Figure 1.**
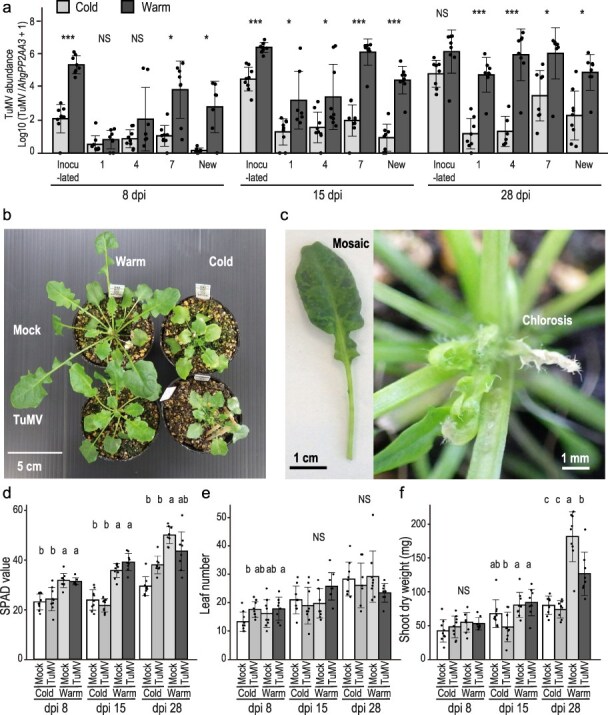
Effects of temperature on growth, symptoms, and virus spread within plants for TuMV- and mock-inoculated *A. halleri* at 8, 15, and 28 dpi under the cold (10°C/5°C, day/night temperatures) and warm (25°C/20°C) conditions. (a) TuMV abundance in leaves at different positions within TuMV-inoculated plants under the cold (gray bars) and warm (black bars) conditions at 8, 15, and 28 dpi (left, middle and right diagrams, respectively). Averages and standard deviations of TuMV amounts (*n* = 7–9) in leaves are shown for each leaf position. Relative leaf positions were determined acropetally from TuMV-inoculated leaves (Position 0), and Position 7 represents the newest leaves at the inoculation (0 dpi). “New” represents the youngest leaves at the time of sampling. Significant differences between cold and warm at the same dpi and positions are indicated by *, **, ***, and NS, corresponding to significance at *P* < .05, .01, .001, and no significant difference at *P* < .05 in *t*-tests with the sequential Bonferroni correction for multiple tests to keep dpi-wise probability. (b) A photograph showing TuMV-inoculated and mock-inoculated plants at 28 dpi under warm and cold conditions. (c) Representative symptoms appeared in the TuMV-inoculated plants at 28 dpi under the warm condition. SPAD value (d), leaf number (e), and shoot dry weight (f) for TuMV- and mock-inoculated plants at 8, 15, and 28 dpi under the cold and warm conditions. Averages and standard deviations are shown for (*n* = 9 and 7–9, for TuMV- and mock-inoculated plants, respectively). Different letters in the diagrams represent statistical significance between four combinations of temperature and virus inoculation treatments within each dpi (*P *< .05, Tukey–Kramer multiple comparison test). NS indicates no significant difference.

To examine the effect of temperature on TuMV accumulation and host growth, the virus was inoculated to uninfected *A. halleri* plants and incubated under warm (25°C/20°C, day/night at 12-h day length) and cold (10°C/5°C, day/night at 12-h day length) conditions. A buffer solution without TuMV was used as a mock-inoculated control. Inoculations were performed on the ninth and 10th leaves. At 8, 15, and 28 dpi, TuMV amounts were quantified for the two inoculated leaves (pooled), the first, fourth, and seventh upper leaves from the inoculated leaves, and a new leaf (the second-youngest leaf at each sampling date that emerged after inoculation) using qPCR. Viral propagation in inoculated leaves and systemic spread to the upper leaves progressed faster under warm conditions than under cold conditions (Fig. 1a). In the inoculated leaves, TuMV abundance increased with time after inoculation (from 8 to 28 dpi) and was significantly higher in the warm treatment than in the cold treatment at 8 and 15 dpi (Fig. 1a). At 28 dpi, TuMV abundance in the inoculated leaves was near and at complete saturation levels under cold and warm conditions, respectively, and became nonsignificant between conditions. In the upper leaves, significant differences in TuMV abundance between the two conditions were found in the seventh leaf at 8 dpi and in all upper leaves at 15 and 28 dpi (Fig. 1a). High viral abundance was continually observed in new leaves at 8, 15, and 28 dpi under the warm conditions (Fig. 1a). Overall, TuMV increment occurred first in the inoculated leaves, followed by the top leaves (Position 7), lower leaves (Positions 1 and 4), and new leaves, although the rate of TuMV increment was slower in the cold treatment.

TuMV inoculation resulted in smaller plant size than the mock-inoculated plants at the warm temperature at 28 dpi (Fig. 1b). Under the cold conditions, plant size was smaller than that in the warm, and no difference was found between TuMV- and mock-inoculated plants at 28 dpi (Fig. 1b). Under the warm conditions, no visible symptoms were observed at 8 dpi. However, mosaic symptoms appeared on newly emerged leaves in some TuMV-inoculated plants at 15 dpi. At 28 dpi, mosaic symptoms were observed in most TuMV-inoculated plants, and a few plants showed chlorosis on newly emerged leaves and leaf rolling on mature leaves (Fig. 1c). No necrosis was observed at least up to 28 dpi. No symptoms were observed under cold conditions. The SPAD value, an index of chlorophyll content, was significantly higher in the warm throughout the experiment, and no statistically significant effect of viral inoculation was detected (Fig. 1d). The initial difference in leaf number at 8 dpi disappeared at 15 and 28 dpi, and no effect of viral inoculation was observed (Fig. 1e). Shoot dry weight increased in the warm compared to the cold at 15 and 28 dpi, and clear growth suppression by virus inoculation was detected in the warm but not in the cold at 28 dpi (Fig. 1f).

### Overall responses of the host transcriptome in cold and warm

To evaluate host responses, we performed transcriptome analyses using RNA-seq of inoculated and systemic leaves (fifth and sixth extant leaves from the inoculated leaves) at 8, 15, and 28 dpi. We excluded the systemic leaves at 8 and 15 dpi under cold conditions from the analyses because no viral accumulation was detected. In total, we obtained RNA-seq data for 10 dpi–position–temperature combinations.

We performed permutational multivariate analysis of variance (PERMANOVA) on the host transcriptome of inoculated leaves to evaluate the overall effects of TuMV inoculation and temperature. Significant effects of TuMV, temperature, and dpi on the host transcriptome were detected (Supplementary Table S1; *R*^2^ = 0.04, 0.19, and 0.07, *F* = 6.37, 27.8, and 11.0, and *P* < .001, .001, and .001 for TuMV, temperature, and dpi, respectively). In the analysis for each dpi, significant effects of TuMV were detected at all time points (Supplementary Table S1, *R*^2^ = 0.09, 0.06, and 0.08; *F *= 3.69, 2.90, and 3.02; and *P* = .007, .009, and .005 at 8, 15, and 28 dpi, respectively), and significant effects of temperature were detected at 8 and 15 dpi (Supplementary Table S1, *R*^2^ = 0.09, 0.07, and 0.04; *F *= 3.74, 2.66, and 1.56; *P* = .006, .011, and .137 at 8, 15, and 28 dpi, respectively).

We then visualized TuMV and temperature effects by principal component analysis (PCA) using all samples of inoculated leaves to determine whether host responses to TuMV inoculation differed between cold and warm conditions at the whole-transcriptome level (Supplementary Fig. S1a–d). The first three principal component axes (PC1, PC2, and PC3) explained 44.0% of the variation in the transcriptome. A clear separation between warm and cold conditions was observed along PC1 (Supplementary Fig. S1a). Responses to TuMV inoculation in the warm were detected along PC2 (Supplementary Fig. S1a and b), whereas those in the cold were detected along PC3 (Supplementary Fig. S1c and d). Although the mock-inoculated samples remained in the same region of the PC1–PC2 space (upper-left region), the TuMV-inoculated leaves showed greater variation along PC2 at 15 and 28 dpi (Supplementary Fig. S1b). Under cold conditions, the mock- and TuMV-inoculated samples were most clearly separated along PC3 at 8 dpi and the separation became less clear at 15 and 28 dpi (Supplementary Fig. S1d). Host responses to TuMV infection under warm and cold conditions appeared to be distinct at the whole-transcriptome level, as their responses were observed along different PC axes.

Because temperature had the most significant effect on the transcriptome, we first examined the difference in host gene expression between warm and cold conditions by examining DEGs in the mock-inoculated control. Upregulated and downregulated DEGs were defined as those with higher and lower gene expression levels, respectively, under cold conditions than under warm conditions. In mock-inoculated leaves, the number of DEGs was highest at 8 dpi (total 7064 with up/down = 3648/3417) and then gradually decreased at 15 dpi (total 6122 with up/down = 3079/3043) and 28 dpi (total 4151 with up/down = 1865/2286) (Supplementary Fig. S2a). The number of DEGs in the systemic leaves (the fifth and sixth leaves upper from the inoculated leaves) at 28 dpi was similar to that in the mock-inoculated leaves at 28 dpi (total 4331 with up/down = 2195/2136) (Supplementary Fig. S2b). In the temperature responses of uninfected host plants, cold-downregulated DEGs were mainly enriched by gene ontology (GO) related to host translation activity and carbon assimilation, such as translation, ribosome assembly, rRNA processing, and photosynthesis (Supplementary Fig. S2c). In contrast, cold-upregulated DEGs were mainly enriched in GOs related to defense, such as defense response to bacterium, response to chitin, and cellular response to hypoxia (Supplementary Fig. S2c).

**Figure 2.**
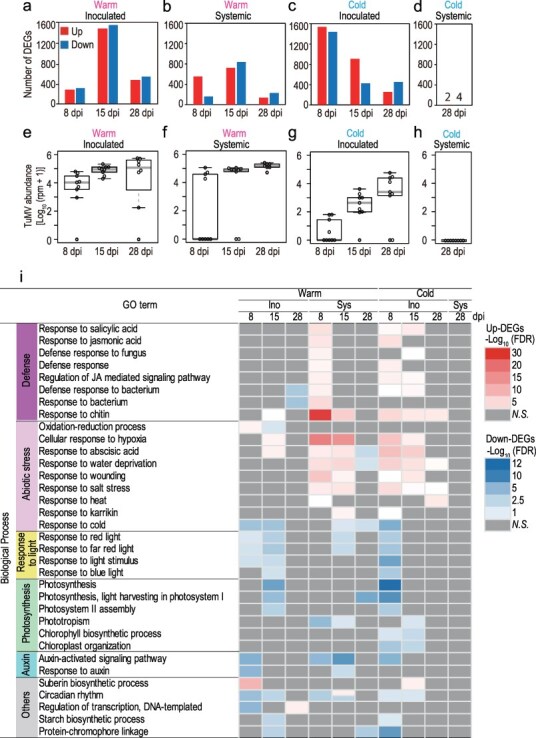
Analyses on DEGs between TuMV- and mock-inoculated plants at different dpi, leaf position, and temperature combinations. Number of DEGs at 8, 15, and 28 dpi for the inoculated and systemic leaves in the warm (a and b) and in the cold (c and d). Upregulated and downregulated DEGs represent those with higher and lower gene expression in the TuMV-inoculated leaves than in the mock-inoculated leaves, respectively. For the systemic leaves in the cold, transcriptome (RNA-seq) analyses were performed only at 28 dpi. Virus accumulation level for the transcriptome samples calculated from TuMV reads included in the RNA-seq data at 8, 15, and 28 dpi for the inoculated and systemic leaves in the warm (e and f) and in the cold (g and h). (i) A heatmap showing the results of GO enrichment analyses on DEGs between TuMV-inoculated and mock-inoculated plants for the 10 dpi–position–temperature combinations. The results of 34 GOs in biological processes that were significant (false discovery rate [FDR] < 0.05) at multiple times are listed. We performed GO analysis separately for upregulated and downregulated DEGs, and there were no cases where the same GO terms were enriched for upregulation and downregulation simultaneously, except for a single case. Therefore, each cell was colored when either upregulated or downregulated DEGs were enriched in the corresponding GO. If the GO is enriched in both upregulated and downregulated DEGs, the cell was divided into two to accommodate both red and blue colors. Gray color represents no enrichment of the corresponding GO.

To elucidate how the transcriptome response to viral infection differed between cold and warm conditions, DEGs were identified by comparing gene expression between TuMV- and mock-inoculated plants. Here, we designated the upregulated and downregulated DEGs as those with higher and lower gene expression, respectively, in TuMV-inoculated leaves than in the mock-inoculated leaves. In the warm treatment, the number of DEGs in the inoculated leaves peaked at 15 dpi (total 3017 with up/down = 1470/1547). The total numbers of DEGs were 570 (up/down = 304/266) and 988 (up/down = 531/457) at 8 and 28 dpi, respectively (Fig. 2a). The number of DEGs for systemic leaves in the warm also peaked at 15 dpi, and 713 (up/down = 554/159), 1565 (725/840), and 366 (137/229) DEGs were detected at 8, 15, and 28 dpi, respectively (Fig. 2b). In contrast, in the cold treatment, the highest number of DEGs was observed at 8 dpi (total 2972 with up/down = 1536/1436). The total number of DEGs gradually decreased to 1328 (up/down = 904/424) at 15 dpi and 704 (up/down = 254/450) at 28 dpi in the inoculated leaves (Fig. 2c). For systemic leaves in the cold treatment, transcriptome analysis was performed only at 28 dpi when six DEGs were detected (Fig. 2d, Supplementary Data 1).

We quantified the level of TuMV accumulation in the RNA-seq-conducted leaves from the RNA-seq reads because they contained both the mRNA and TuMV genome with a polyadenylation (poly-A) tail (Fig. 2e–h, note that quantification of TuMV in Fig. 1a was conducted by more sensitive qPCR for different leaves). The number of DEGs was not necessarily explained by the virus accumulation levels. When DEGs peaked in the warm for inoculated and systemic leaves at 15 dpi, virus accumulation almost saturated (Fig. 2e and f), whereas the number of DEGs was highest when virus accumulation was still low at 8 dpi for the inoculated leaves in the cold (Fig. 2g). The small number of DEGs (=6) in the cold systemic leaves at 28 dpi was due to the low level of TuMV, which was below the detection limit of RNA-seq (Fig. 2h).

### GO enrichment analysis for all experimental combinations and for K-means clusters

We performed GO enrichment analyses for each of the 10 dpi–position–temperature combinations because most DEGs were transient and unique to each combination. We performed GO analyses separately for the upregulated and downregulated DEGs, and no cases occurred in which the same GO terms were simultaneously enriched for upregulation and downregulation, except for one case. In total, 90 biological process GOs were enriched at least once (40 and 57 upregulated and downregulated GOs, respectively; Supplementary Fig. S3), of which 34 GOs became significant in multiple combinations (Fig. 2i).

GOs that were significantly enriched in multiple combinations included defense, abiotic stress, light response, and photosynthesis-related GOs (Fig. 2i). Enrichment of defense-related GOs in the upregulated DEGs was often shared between the systemic leaves in the warm treatment and the inoculated leaves in the cold treatment at earlier timings (Fig. 2i). The enrichment of abiotic stress-related GOs in the upregulated DEGs was shared between systemic leaves under warm conditions and inoculated leaves under cold conditions at 8 and 15 dpi for those related to hypoxia, drought, heat, and wounding (Fig. 2i). GOs related to cold and light responses were frequently enriched in downregulated DEGs in inoculated leaves at 8 and 15 dpi and in systemic leaves at 15 dpi under warm conditions and in inoculated leaves at 8 dpi under cold conditions (Fig. 2i). Enrichment of photosynthesis-related GOs terms was detected in the downregulated DEGs in inoculated leaves at 15 dpi under warm conditions and at 8 dpi under cold conditions (Fig. 2i). Other characteristic GOs were auxin-related, which were often enriched in the downregulated GOs under warm conditions (Fig. 2i). Overall, defense- and abiotic stress-related GOs were enriched in the upregulated DEGs at similar time points in each leaf position, i.e. at early time points (8 and 15 dpi) of systemic leaves in the warm and inoculated leaves in the cold. In contrast, in downregulated DEGs, photosynthesis, auxin, and response light-related GOs were enriched in inoculated leaves in the warm treatment, including in the late stage (28 dpi), but not at 28 dpi of the cold treatment in systemic leaves.

Among the remaining GOs (single-time-enriched GOs) in the biological processes, the top three highly enriched upregulated GOs were rRNA processing (cold, inoculated, 8 dpi, −log_10_FDR = 9.55), intracellular protein transport (warm, inoculated, 15 dpi, −log_10_FDR = 8.96), and response to bacterium (warm, systemic, 8 dpi, −log_10_FDR = 5.00) (Supplementary Fig. S3). The top three highly enriched downregulated GOs were defense response by callose deposition in cell wall (warm, inoculated, 28 dpi, −log_10_FDR = 11.14), reductive pentose phosphate cycle (cold, inoculated, 8 dpi, −log_10_FDR = 5.74), and gluconeogenesis (cold, inoculated, 8 dpi, −log_10_FDR = 5.23) (Supplementary Fig. S3).

To incorporate quantitative information on gene expression across the entire experiment, we performed *K*-means clustering using all the DEGs (Supplementary Fig. S4). We identified six clusters and performed a GO enrichment analysis. Similar to the aforementioned analyses in Fig. 2i, the overall enrichment of defense-, abiotic stress-, light response-, and photosynthesis-related GOs was detected (Supplementary Fig. S4). Cluster 1 was characterized by prominent upregulation following viral infection, specifically in cold-inoculated leaves, and was enriched in GOs related to abiotic stress. Genes in Clusters 3 and 4 shared a similar pattern of upregulation with dpi in mock-inoculated leaves, and these changes were accelerated by TuMV inoculation. Both clusters were enriched in abiotic stress and defense. Cluster 3 showed a stronger effect of TuMV infection, characterized by high expression in the warm in general, and was enriched in GOs related to leaf senescence and SA response. Cluster 6 genes were downregulated by viral infection, and the enriched GO terms included those related to photosynthesis and auxin signaling. These results allowed us to characterize the responses of some of the DEGs quantitatively. For example, some DEGs tended to retain similar response levels throughout the dpi, as observed in the patterns in Cluster 1 (Supplementary Fig. S4). As in Clusters 3, 4, 5, and 6, the expression of many genes changed in response to temperature, dpi, leaf positions, or their combinations, even in mock-inoculated plants, and TuMV infection–modified gene expression, maintaining basal responses in most cases (Supplementary Fig. S4). In the following sections, the gene names of *A. halleri* were determined by adding *Ahg* to the names of homologous genes in *A. thaliana* (we used gene ID only when the homologs had no name).

### Defense and other related genes

**Figure 3.**
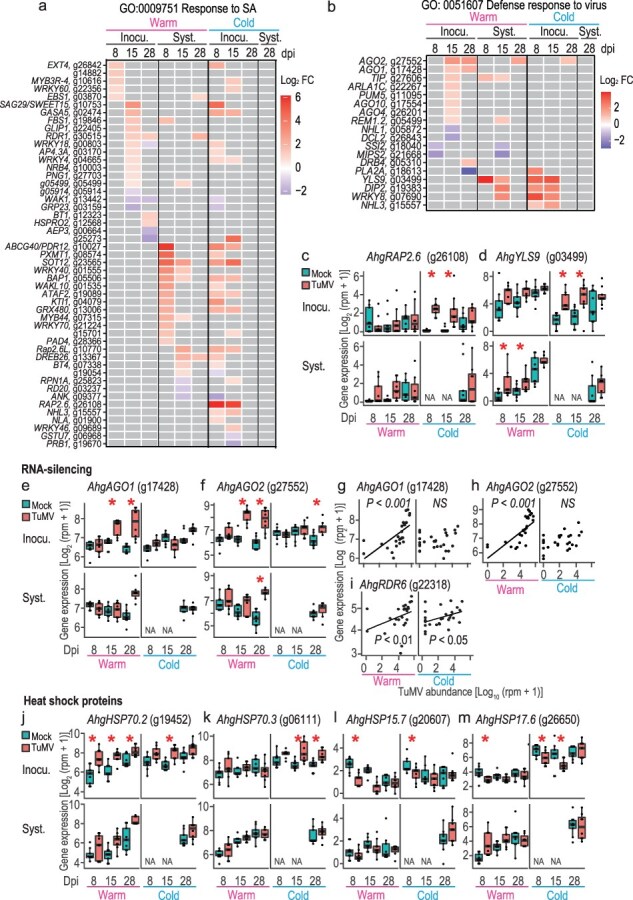
Gene expression patterns of DEGs between TuMV- and mock-inoculated plants related to defense and virus response. Heatmaps showing at which dpi–position–temperature combinations the genes were detected as DEGs for the “response to SA” GO (a) and “defense response to virus” GO (b). Gray color represents no significant difference between virus+ and virus− leaves (EdgeR, FDR < 0.05). (c) Gene expression in all samples for *AhgRAP2.6*, an example of a DEG selected from (a). (d) Gene expression in all samples for *AhgYLS9*, an example of a DEG selected from (b). Gene expression in all samples for *AhgAGO1* (e) and *AhgAGO2* (f), genes representing RNA silencing. Dependence of gene expression on TuMV abundance of inoculated leaves in the warm and cold for *AhgAGO1* (g), *AhgAGO2* (h), and *AhgRDR6* (i). Data from inoculated leaves at 8, 15, and 28 dpi were used for each temperature condition. Regression lines and significance levels (*P*) are shown when the regression is statistically significant. Gene expressions in all samples for representative HSPs were detected as DEGs, *AhgHSP70.2* (j), *AhgHSP70.3* (k), *AhgHSP15.7* (l), and *AhgHSP17.6* (m). In (c–f) and (j–m), the upper and lower columns represent the results from inoculated and systemic leaves, respectively. NA indicates no data available. Asterisks represent significant difference between virus+ and virus− leaves (EdgeR, FDR < 0.05).

We examined the responses of genes that were included in three GO terms related to virus defense, i.e. “response to SA,” “defense response to virus,” and “response to virus” (Fig. 3, Supplementary Fig. S5). Regarding the SA response, the genes upregulated in the warm treatments were largely different between the inoculated and systemic leaves (Fig. 3a). Genes in both groups were upregulated in the inoculated leaves under cold conditions; however, the similarity between systemic leaves under warm conditions and inoculated leaves under cold conditions was more pronounced (Fig. 3a). Among the WRKY transcription factors, *AhgWRKY18* and *AhgWRKY60* were upregulated in inoculated leaves under warm and cold conditions, whereas *AhgWRKY40* was upregulated only in systemic leaves under warm conditions (Fig. 3a). The homologs of these three WRKYs are coexpressed in response to pathogen attack and abiotic stress in *A. thaliana*. WRKY40 has been reported to act as a repressor of the ABA response by antagonizing WRKY18 and WRKY60 (Xu et al. 2006, Chen et al. 2010). A gene encording transcription factor, *AhgRELATED TO AP2.6* (*AhgRAP2.6*), was detected as a highly upregulated DEG in the inoculated leaves in the cold (Fig. 3a and c). This gene was strongly repressed in mock-inoculated leaves at 8 and 15 dpi and then upregulated at 28 dpi in the cold treatment, whereas it was induced earlier by TuMV inoculation (Fig. 3c).

For the “defense response to virus” GO, most genes were detected as upregulated DEGs (Fig. 3b). Genes encoding ARGONAUTEs, representative RNA silencing proteins (*AhgAGO1, AhgAGO2, AhgAGO4*, and *AhgAGO10*), were upregulated at 15 and 28 dpi (Fig. 3b). For example, *AhgAGO1* was significantly upregulated in inoculated leaves at 15 and 28 dpi (Fig. 3e). *AhgAGO2* was detected as an upregulated DEG in inoculated leaves at 15 and 28 dpi, systemic leaves at 28 dpi in the warm treatment, and inoculated leaves at 28 dpi in the cold treatment (Fig. 3f). Under warm conditions, the expressions of *AhgAGO1*, *AhgAGO2*, and *AhgRDR6* positively correlated with TuMV abundance in the inoculated leaves (Fig. 3g–i). These correlations were weakened under cold conditions, and only *AhgRDR6* positively correlated with TuMV abundance (Fig. 3g–i). *AhgREM1.2* was upregulated at 15 dpi in inoculated and systemic leaves under warm conditions (Fig. 3b), and the *A. thaliana* homolog of this gene has been reported to be a negative regulator of the cell-to-cell movement of TuMV (Cheng et al. 2019). In the cold treatment, *AhgYLS9* (Fig. 3d), a PR gene that suppresses CMV infection (Elsharkawy et al. 2022), and three other genes (*AhgDIP2, AhgWRKY8*, and *AhgNHL3*) were strongly upregulated in the inoculated leaves. All of them, except *AhgNHL3*, were also upregulated in the systemic leaves in the warm treatment.

For the “response to virus” GO, most DEGs were detected as upregulated ones (Supplementary Fig. S5). These included a series of genes encoding heat shock protein 70 (HSP70), such as *AhgHEAT SHOCK PROTEIN 70.2* (*AhgHSP70.2*) and *AhgHSP70.4* (Fig. 3j and k). It has been reported that *HSP70* is upregulated in *A. thaliana* by many viruses such as TuMV, CMV, oilseed rape mosaic virus, potato virus X, and turnip vein clearing virus (Whitham et al. 2003). Some small HSP genes, such as *AhgHSP15.7* and *AhgHSP17.6*, were downregulated (Fig. 3l and m).

### Auxin-activated signaling pathway

**Figure 4.**
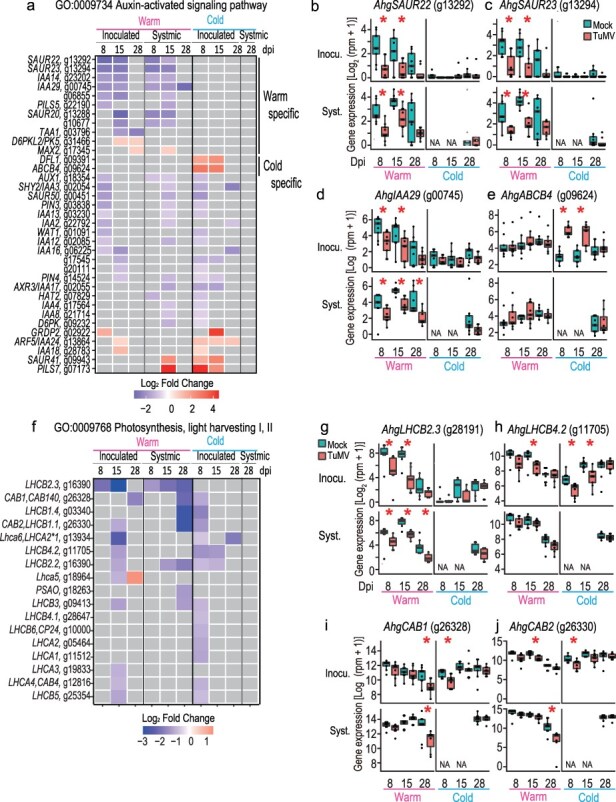
Gene expression of DEGs between TuMV- and mock-inoculated plants related to auxin response and photosynthesis. (a) A heatmap showing in which dpi–position–temperature combinations the genes were detected as DEGs for the “auxin-activated signaling pathway” GO. Gene expression in all samples for *AhgSAUR22* (b), *AhgSAUR23* (c), *AhgIAA29* (d), and *AhgABCB4* (e), examples of DEGs selected from (a). (f) A heatmap showing in which dpi–position–temperature combinations the genes were detected as DEGs for the “photosynthesis, light-harvesting I, II” GO. Gene expression in all samples for *AhgLHCB2.3* (g), *AhgLHCB4.2* (h), *AhgCAB1* (i), and *AhgCAB2* (j), examples of DEGs selected from (f). In heatmaps (a and f), gray color represents no significant difference between virus+ and virus− leaves (EdgeR, FDR < 0.05). In (b–e) and (g–j), the upper and lower columns represent the results from inoculated and systemic leaves, respectively. NA indicates no data available. Asterisks represent the significant difference between virus+ and virus− leaves (EdgeR, FDR < 0.05).

It turned out that 83 out of 172 genes belonging to the auxin-activated signaling pathway, which is the coordinator of plant growth and development, altered their expression at least once in response to TuMV inoculation in all dpi–position–temperature combinations (Supplementary Table S2). Among them, 35 genes were consistently upregulated or downregulated on multiple occasions, and 11 and 2 DEGs were warm- and cold-specific, respectively (Fig. 4a). Among the warm-specific DEGs, most were downregulated by TuMV infection (Fig. 4a). For example, the small auxin-upregulated RNA (SAUR) genes, *AhgSAUR22* and *AhgSAUR23*, were induced under warm conditions, whereas they were repressed by TuMV inoculation at 8 and 15 dpi in the inoculated and systemic leaves (Fig. 4b and c). These genes were not expressed under cold conditions regardless of TuMV infection (Fig. 4b and c). *AhgINDOLE-3-ACETIC ACID 29* (*AhgIAA29*) showed suppression patterns similar to *AhgSAUR22* and *AhgSAUR23* (Fig. 4d). Growth deficiency was caused by TuMV infection under warm conditions and was also caused by the cold irrespective of the presence/absence of TuMV infection (Fig. 1a and c). This corresponded to the expression patterns of these genes. Two genes, *AhgDWARF IN LIGHT 1* (*AhgDFL1/GH3.6*) and *AhgATP-BINDING CASSETTE B4 (AhgABCB4*), were upregulated cold-specifically (Fig. 4a and e). The homologs of these genes are positive regulators of meristem size and IAA transport in *A. thaliana* roots (Kubeš et al. 2012, Pierdonati et al. 2019, Wang et al. 2023).

### Photosynthesis-related genes

Photosynthesis-related DEGs were mostly downregulated, presumably representing host damage caused by TuMV infection (Fig. 4f). However, the situation at 28 dpi differed between warm and cold conditions. Under warm conditions, most DEGs were significantly downregulated at 15 dpi in inoculated leaves and 28 dpi in systemic leaves (Fig. 4f). Under cold conditions, the significant downregulation of photosynthesis-related DEGs mainly occurred at 8 dpi (Fig. 4f), when the level of virus accumulation in the inoculated leaves was still moderate (Fig. 1a). Expression of most of the genes belonging to the “light-harvesting II” GO, such as *AhgLIGHT-HARVESTING CHLOROPHYLL B-BINDING 2.3* (*AhgLHCB2.3*) and *AhgLHCB4.2*, decreased from 8 to 28 dpi even in the mock-inoculated plants in the warm, and the expressions were more strongly suppressed in TuMV-inoculated plants (Fig. 4g and h). In contrast, in the cold treatment, gene expression was low at 8 dpi but increased at 15 and 28 dpi in the mock-inoculated plants (Fig. 4g and h). Downregulation of photosynthesis-related genes in the inoculated leaves occurred in 13 genes at 8 dpi, but the gene expression levels recovered by 28 dpi for 12 genes, except for *AhgLHCA6*. TuMV infection significantly repressed gene expression at 8 and 15 dpi for *AhgLHCB4.2* in the inoculated leaves under cold conditions, whereas expression in TuMV-inoculated plants increased and reached a level equivalent to that in mock-inoculated plants at 28 dpi. For *AhgCHLOROPHYLL A/B BINDING PROTEIN 1* (*AhgCAB1/LHCB1.3*) and *AhgCAB2/LHCB1.1* genes, no such contrasting pattern was observed in the mock-inoculated leaves between warm and cold conditions; however, repression of gene expression by TuMV infection was detected at 15 and 28 dpi under warm conditions and 8 dpi under cold conditions (Fig. 4i and j).

### Temperature-specific DEGs

**Figure 5.**
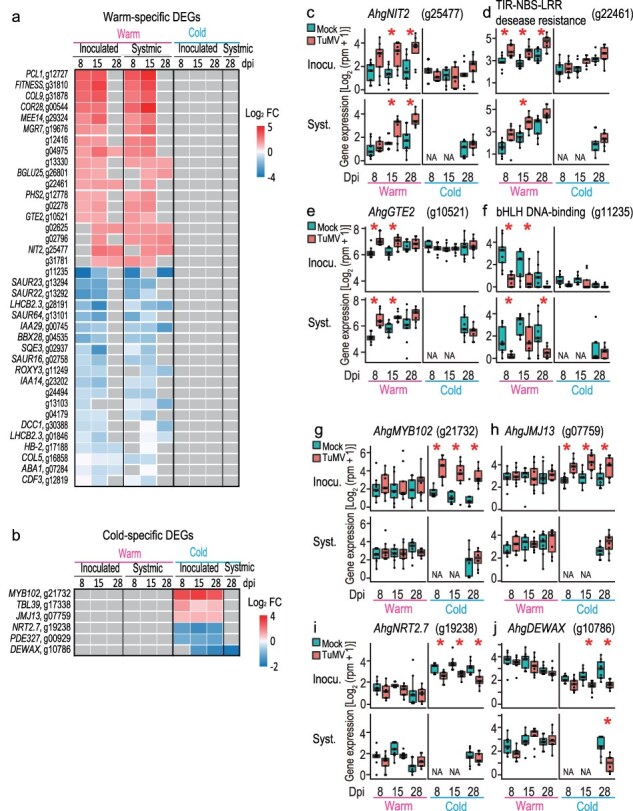
Temperature-specific DEGs between TuMV- and mock-inoculated plants. Heatmaps showing at which dpi–position–temperature combinations the genes were detected as warm-specific DEGs (a) and cold-specific DEGs (b). Gene expression patterns for *AhgNIT2* (c), **a** TIR-NBS-LLR disease resistance gene (g22461) (d), *AhgGTE2* (e), and a bHLH DNA-binding gene (g11235) (f), examples of DEGs selected from warm-specific DEGs. Gene expression patterns for *AhgMYB102* (g), *AhgJMJ13* (h), *AhgNRT2.7* (i), and *AhgDEWAX* (j) are examples of DEGs selected from cold-specific DEGs. In heatmaps (a and b), gray color represents no significant difference between virus+ and virus− leaves (EdgeR, FDR < 0.05). In (c–j), the upper and lower columns represent the results from inoculated and systemic leaves, respectively. NA indicates no data available. Asterisks represent significant difference between virus+ and virus− leaves (EdgeR, FDR < 0.05).

To list genes that responded to TuMV infection in a temperature-specific manner, we defined warm- and cold-specific DEGs as genes detected as DEGs in at least four and three combinations under warm and cold conditions, respectively (more than half of all dpi–position combinations). Genes with inconsistent response directions were excluded. The numbers of warm- and cold-specific DEGs were 38 (up/down = 18/20) and 6 (up/down = 3/3), respectively (Fig. 5a and b).

Warm-specific downregulated DEGs included auxin-related genes (*AhgSUAR16, 22, 23*, and *64* and *AhgIAA14* and *29*, Fig. 4b–d) and *AhgLHCB2.3* (Fig. 4g), which we have treated in the “Auxin-activated signaling pathway” and “Photosynthesis-related genes” section, respectively. In addition, one warm-specific upregulated DEG was the auxin biosynthetic gene *AhgNIT2* (g25477) (Bartling et al. 1992, Normanly et al. 1997, Lehmann et al. 2017), and its expression pattern was similar to that of other auxin-related genes (Fig. 5c). Other examples of warm-specific upregulated DEGs included a homolog encoding a disease resistance protein, one of the TIR-NBS-LRR class genes (g22461), and the transcription factor *AhgGTE2* (g10521) which is also considered a chromatin-remodeling factor (Farrona et al. 2007) (Fig. 5d and e). An example of a warm-specific downregulated DEG is the bHLH DNA-binding protein gene, which is a homolog of AT3G21330 (g11235) (Fig. 5f).

Cold-specific upregulated DEGs included *AhgMYB102* (g21732) and *AhgJMJ13* (g07759), and cold-specific downregulated DEGs included *AhgNRT2.7* (g19238) and *AhgDEWAX* (g10786) (Fig. 5g–j). MYB102, a transcription factor, is known to increase plant susceptibility to aphids by reducing callose deposition (Zhu et al. 2018), and JMJ13, an H3K27me3 demethylase, has been reported to act as a temperature-dependent flowering repressor in *A. thaliana* (Zheng et al. 2019). DEWAX is a negative regulator of cuticular-wax biosynthesis (Suh and Go 2014).

### Constant DEGs

**Figure 6.**
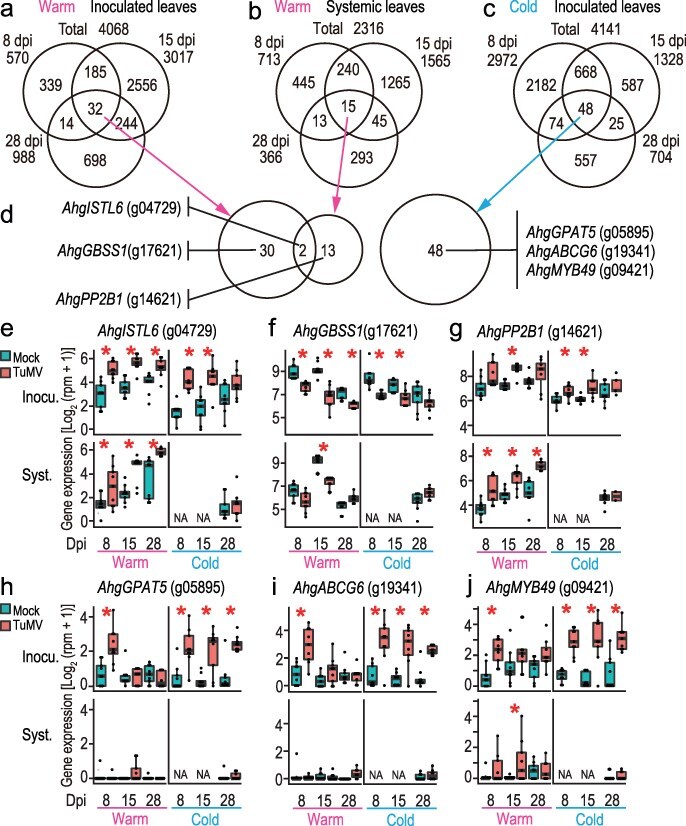
DEGs detected constantly across time points (constant DEGs) in the warm and cold. Venn diagrams for DEGs at 8, 15, and 28 dpi in the inoculated (a) and systemic leaves (b) in the warm (25°C/20°C) and in the inoculated leaves in the cold (15°C/10°C) (c) to show that 32, 15, and 48 genes are constant DEGs, respectively. (d) Venn diagrams showing the overlap between constant DEGs in the inoculated and systemic leaves in the warm and in the inoculated leaves in the cold. There was no overlap in constant DEGs between the warm and cold DEG. Representative DEGs belonging to each region of the Venn diagram in (d) are listed. (e–j) Gene expression in all samples for representative constant DEGs, *AhgISTL6* , a TIR-NBS-LRR gene (e), *AhgGBSS1* (f), *AhgPP2-B1* (g), *AhgGPAT5* (h), *AhgABCG6* (i), and *AhgMYB49* (j). In (e–j), the upper and lower columns represent the results from inoculated and systemic leaves, respectively. NA indicates no data available. Asterisks represent significant difference between virus+ and virus− leaves (EdgeR, FDR < 0.05).

To determine whether the gene expression responses to TuMV were transient, we examined the overlap between DEGs at 8, 15, and 28 dpi in inoculated and systemic leaves under warm conditions (Fig. 6a and b) and in inoculated leaves under cold conditions (Fig. 6c). Most of the DEGs were found to be transient, and DEGs unique to one or two consecutive sampling dates accounted for 98.9% (4022), 98.8% (2288), and 97.1% (3019) of the 4068, 2316, and 4141 total DEGs for inoculated and systemic leaves in the warm and inoculated leaves in the cold, respectively (Fig. 6a–c). Constant DEGs were detected across all time points, including 32, 15, and 48 genes for inoculated and systemic leaves under warm conditions and inoculated leaves under cold conditions, respectively (Fig. 6d, Supplementary Table S3). Among these constant DEGs, five, six, and five were temperature-specific (described in the previous section), respectively.

Here, we describe the constant DEGs, excluding temperature-specific DEGs. One of the two overlapping constant DEGs between the inoculated and systemic leaves under the warm condition was *AhgIST1-LIKE6* (*AhgISTL6*, g04729), which was upregulated under all conditions except for the inoculated and systemic leaves at 28 dpi under the cold condition (Fig. 6e). In a previous field study, the TuMV–*A. halleri* interactions, this gene was detected as a DEG in both spring and autumn (Honjo et al. 2020). An example of constant DEGs for inoculated leaves in the warm was *AhgHSP70.2*, which was upregulated by TuMV inoculation and described earlier in the “Defense and other related genes” section (Fig. 3j). *AhgGRANULE BOUND STARCH SYNTHASE 1* (*AhgGBSS1*), whose homolog is responsible for amylose synthesis in the storage organs of *A. thaliana* (Seung et al. 2015), was a downregulated constant DEG in the inoculated leaves in the warm treatment (Fig. 6f). Constant DEGs in systemic leaves included *AhgPHLOEM PROTEIN 2-B1* (*AhgPP2-B1*; Fig. 6g), *AhgIAA29* (Fig. 4e), and *AhgLHCB2.3* (Fig. 4f). Among these DEGs, *AhgISTL6* and *AhgPP2-B1* are among the three genes commonly upregulated in the spring and autumn equinoxes in a natural environment (Honjo et al. 2020), suggesting that they are not transient and are upregulated in response to the presence of TuMV over a wide range of temperatures.

The constant DEGs for the inoculated leaves in the cold were enriched by the “biosynthetic process of suberin” GO (Supplementary Table S4), which is a lipid-phenolic biopolyester deposited in the cell walls of certain plant boundary tissue layers. Three genes related to suberin biosynthesis were detected as DEGs, i.e. *AhgGLYCEROL-3-PHOSPHATE sn-2-ACYLTRANSFERASE 5* (*AhgGPAT5*, Fig. 6h), *AhgATP-BINDING CASSETTE G6* (*AhgABCG6*, Fig. 6i), and *AhgRWP1*. These three genes showed similar regulatory patterns and were upregulated in TuMV-inoculated leaves constantly under cold conditions and transiently at 8 dpi under warm conditions (Fig. 6h and i). The gene encoding the transcription factor *AhgMYB49* showed a similar expression pattern (Fig. 6j). MYB49 promotes the expression of genes involved in suberin, cutin, and wax biosynthesis in *A. thaliana* (Zhang et al. 2020). Further studies evaluating the outcomes of these responses should include the quantification of suberin, cutin, and callose deposition after TuMV inoculation under different temperature conditions.

### Network analysis

Using all transcriptome datasets, we examined the coexpression relationships between genes by applying network analysis to identify the modules and hub genes associated with TuMV infection. The most significantly upregulated and downregulated modules were dark olivegreen (correlation with the presence or absence of TuMV infection 0.40, *P* < 4.56 × 10^−8^) and saddlebrown (correlation −0.36, *P* < 1.26 × 10^−6^), respectively (Supplementary Fig. S6).

**Figure 7.**
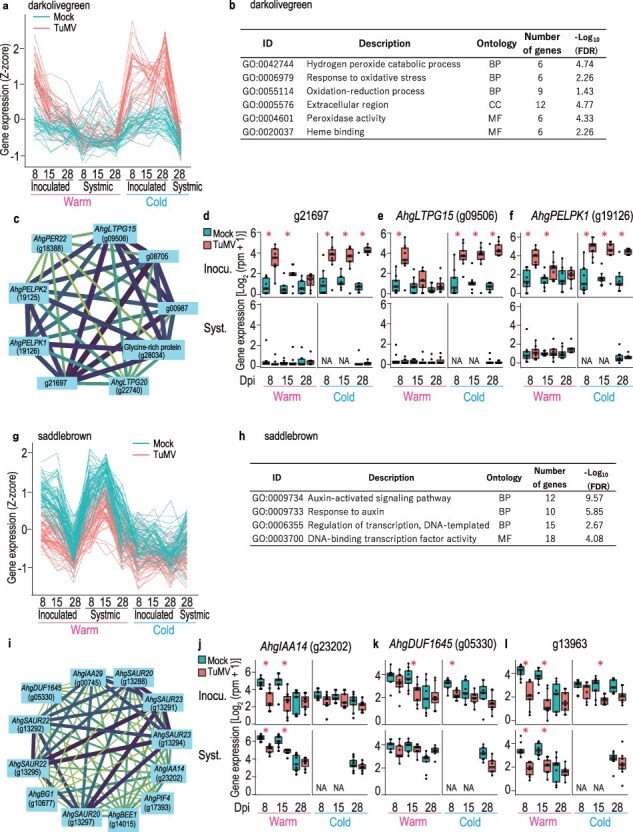
Results of the WGCNA performed on all expressed genes. (a) Gene expression pattern of all genes (37 genes) in the darkolivegreen module. (b) Results of GO enrichment analysis for genes in the darkolivegreen module. (c) Networks of the top hub genes with the highest number of connections and their connectivity to the other genes for the darkolivegreen module. Gene expression in all samples for g21697 (d), *AhgLTPG15* (e), and *AhgPELPK1* (f), examples of hub genes selected from the darkolivegreen module. (g) Gene expression pattern of all genes (65 genes) in the saddlebrown module. (h) Results of GO enrichment analysis for genes in the saddlebrown module. (i) Networks of the top hub genes with the highest number of connections and their connectivity to the other genes for the saddlebrown module. Gene expression in all samples for *AhgIAA14* (j), *AhgDUF1645* (k), and g13963 (l), examples of hub genes selected from the saddlebrown module. In (c) and (i), the width of the lines represents the weight of the connection. In (d–f) and (j–l), the upper and lower columns represent the results from inoculated and systemic leaves, respectively. NA indicates no data available. Asterisks represent significant difference between virus+ and virus− leaves (EdgeR, FDR < 0.05).

The expression pattern in the darkolivegreen module was characterized by upregulation in response to TuMV infection in inoculated leaves at an early time point in the warm treatment and by continuous upregulation across time points in the cold treatment (Fig. 7a). In this module, GOs related to reactive oxygen species (ROS) responses, including hydrogen peroxide catabolic process, were enriched (Fig. 7b). The top two hub genes identified in the darkolivegreen module were g21697 (AT2G22510 homolog), which encodes a hydroxyproline-rich glycoprotein family, and *AhgLTPG15* (g09506, AT2G48130 homolog), which encodes a bifunctional inhibitor/lipid-transfer protein/seed storage 2S albumin superfamily. The latter has been reported to function in suberin monomer export in the seed coat and enhance seed germination in *A. thaliana* (Lee and Suh 2018). The network of the top nine genes with the highest number of connections in the darkolivegreen module included four genes that encoded hydroxyproline-rich glycoproteins such as *AhgPELPK1* and *AhgPELPK2* (Fig. 7c). The top two hub genes and *AhgPELPK1* showed typical expression patterns in this module (Fig. 7d–f). PELPK1 has been reported to be a positive regulator of seed germination in *A. thaliana* (Rashid and Deyholos 2011).

The expression pattern in the saddlebrown module was characterized by downregulation in response to viral infection at early time points in both inoculated and systemic leaves under warm conditions (Fig. 7g). This module enriched GO terms related to auxin responses, including the auxin-activated signaling pathway GO (Fig. 7h). The top six hub genes in this module comprised three sets of two genes identified as homologs of *SAUR22*, *SAUR23*, and *IAA29: AhgSAUR22* (g13292 and g13295), *AhgSAUR23* (g13291 and g13294), and *AhgIAA29* (g13288 and g13297) (Fig. 7i). At least one of the homologs of each pair was also detected as the representative auxin-related DEGs in the “Auxin-activated signaling pathway” section (Fig. 4b–d), and cold-specific downregulated DEGs in the “Temperature-specific DEGs” section, *AhgIAA14* (g23202, encoding an auxin-responsive protein and a homolog of AT4G14550), *AhgDUF1645* (g05330, a stress response NST1-like protein and a homolog of AT3G62630), and g13963 (a phytochrome kinase substrate–like protein and a homolog of AT1G18810) were detected in this module (Fig. 7j–l).

## Discussion

This study demonstrated that three aspects of virus–host interactions are highly temperature-dependent: viral accumulation, host symptoms, and host transcriptomic responses. Cold conditions (10°C/5°C, 12/12 h) strongly suppressed virus accumulation compared to warm conditions (25°C/20°C, 12/12 h), and it took three times as long to achieve a similar virus distribution within host plants at 28 dpi under the cold conditions as at 8 dpi under the warm conditions. Growth inhibition and leaf symptoms caused by TuMV, such as chlorophyll reduction and chlorosis, were observed under warm conditions but not under cold conditions.

The suppression of viral accumulation by cold can explain the low viral levels during winter in the natural population of *A. halleri*, a perennial evergreen herb reported previously (Honjo et al. 2020). The cold conditions in this study, 10°C/5°C for day/night temperature, were relatively mild compared with those in mid-winter in natural habitats. Our results suggest that viruses inhibit host growth only during the warm season. The negative effects of viral infection on host growth may be minimal not only during winter but also in early spring and late autumn, during which the host can grow in the natural population. Attenuation of growth inhibition during cold seasons may be one of the key mechanisms for the persistent interaction between the virus and the host.

The slow viral accumulation and weak symptoms under cold conditions must be carefully interpreted. This can directly result from lowered host activity due to cold because the virus relies on host activity during its replication. In comparing transcriptomes between warm and cold conditions in uninfected plants (mock-inoculated controls), genes related to translation, rRNA processing, and chloroplast organization were downregulated by cold conditions. Alternatively, a cold-specific host response that slows viral accumulation and weakens the symptoms may exist. Despite slower virus accumulation and weaker symptoms, we observed an equivalent level of the transcriptomic response to viral infection in the cold compared to that under the warm conditions regarding the number of DEGs. The DEGs between the mock and TuMV inoculation groups were largely unique at each temperature and most appeared transiently at various times after inoculation, representing the complex nature of the interactive effects of temperature and viral infection on the host transcriptome. However, some consistently induced genes (constant DEGs) were identified, whose expression was induced by TuMV even after 28 days of inoculation. These genes may affect plants during persistent long-term infections.

RNA-seq analyses provided comprehensive information on the host response to virus infection in warm and cold environments and allowed us to speculate on the candidate mechanisms underlying distinct virus accumulation and host symptoms. However, further validation is required by manipulating candidate genes (e.g. silencing or overexpression) using an effective transgene procedure in self-incompatible *A. halleri*, which is still under development. The use of TuMV–*A. thaliana* system is another strategy for applying transgenic methods and utilizing mutant lines. The discussion in the following sections is subject to the same limitations at this stage of the study.

### Temperature effects on symptom and transcriptome

The most obvious symptom was growth inhibition under warm conditions, which was detected as a lower shoot weight at 28 dpi. However, under cold conditions in which the growth of uninfected plants was suppressed, no further growth inhibition by viral infection was observed (Fig. 1). The auxin signaling pathway was detected as an enriched GO in downregulated DEG at multiple leaf positions and dpi combinations under warm conditions. Because auxins are often associated with plant growth (Zhang et al. 2022), we speculated that the suppression of auxin signaling could be one of the mechanisms of growth inhibition by TuMV infection under warm conditions. Eight of the nine warm-specific downregulated DEGs, including *AhgIAA29, AhgSAUR20, AhgSAUR22*, and *AhgSAUR23*, were suppressed in both mock- and TuMV-inoculated plants in the cold, and the patterns were consistent with observing small plant sizes in the cold, regardless of the presence or absence of the virus. Large-scale disruption of auxin regulation has also been reported in the TMV–*A. thaliana* system, and 30% of the genes transcriptionally altered by TMV infection contain promoters with multiple auxin response elements (Padmanabhan et al. 2005). The interaction between a TMV replicase and an auxin response regulator, phytochrome-associated protein 1 (PAP1/IAA26), has been proposed to underlie the significant disruption of the auxin signaling pathway by TMV infection (Padmanabhan et al. 2005).

Many photosynthesis-related GOs were enriched in the downregulated DEGs under both warm and cold conditions (Fig. 2), and suppressing photosynthetic activity was likely a symptom of TuMV infection. In the cold treatment, downregulation of photosynthesis-related genes occurred at 8 dpi, but gene expression levels were recovered by 28 dpi in 12 out of 13 DEGs. In contrast, at warmer temperatures, photosynthesis-related genes were detected as downregulated DEGs at 15 dpi and remained low at 28 dpi, but by this time, the expression of these genes was also reduced in uninfected plants with aging leaves (Fig. 4). The response pattern observed under cold conditions is similar to that reported in cases of high-stress tolerance, where the stress response at the gene expression level is strong in the early stages and then returns to normal (Yamaguchi-Shinozaki and Shinozaki 2006). A previous study showed that downregulation of the photosynthesis-related gene *NbLHCB3* is involved in the defense against TuMV by inducing ROS production (Qiu et al. 2021). Further experimental validation is required to determine whether the downregulation of photosynthesis-related genes under cold conditions is involved in the defense against viral infection.

Interestingly, we detected cold-specific genes that responded to TuMV infection under cold but not warm conditions. These genes include MYB102, a transcription factor, and JMJ13, a H3K27me3 demethylase that acts as flowering repressors in *A. thaliana* (Zheng et al. 2019). The flowering of *A. halleri* is induced and initiated under long cold exposure in winter (Aikawa et al. 2010, Satake et al. 2013, Nishio et al. 2020). Therefore, future studies should investigate whether TuMV-infected individuals exhibit different flowering responses.

### Temperature effects on virus accumulation

Little is known about the molecular mechanisms underlying temperature-dependent viral replication. HSPs, such as HSP70 and HSP90, are well known to be important for (+) RNA virus replication (Nagy et al. 2011) and are transcriptionally induced by the virus (TuMV; Aparicio et al. 2005). HSP70-3 has been reported to interact with the RNA-dependent RNA polymerase of TuMV (Dufresne et al. 2008). In our study, some genes of the HSP70 family were upregulated under warm and cold conditions, whereas others were upregulated only under cold conditions. In our previous study on a natural population, we did not detect HSP genes as DEGs between infected and uninfected plants (Honjo et al. 2020), but it should be noted that our previous study was not designed to detect transient upregulation after infection.

We also observed the downregulation of homologs of some small HSPs, such as *AhgHSP15.7* and *AhgHSP17.6*, in addition to those upregulated in response to viral infection. There is little information on how small HSPs are involved in virus–plant interactions (Waters and Vierling 2020), and this aspect requires further investigation. Lower TuMV accumulation in the inoculated leaves reflects the suppression of viral replication within the host cell and/or cell-to-cell movement. At 28 dpi, TuMV reached systemic leaves even under cold conditions, presumably triggered by increased TuMV accumulation at the infection site. Our RNA-seq analysis showed no sign that systemic movement efficiency differed between warm and cold conditions.

### Defense response

SA signaling and RNA silencing are the two major defense mechanisms against viral infections. SA serves as an early defense mechanism in response to pathogen attacks (Qin et al. 1994), but the timescales of the RNA silencing response have not been well studied. In our experiments, most SA signaling genes were induced earlier than RNA silencing genes. Most SA response genes were upregulated at early time points during viral propagation after inoculation (8 and 15 dpi) under both warm and cold conditions, whereas the upregulation of key RNA silencing genes (*AhgAGO1* and *AhgAGO2*) occurred relatively later at 15 and 28 dpi, mainly under warm conditions. The expression patterns of RNA silencing genes in the warm, such as *AhgAGO1, AhgAGO2*, and *AhgRDR6*, can be explained by the level of viral accumulation, as the expression of these genes showed significant positive correlations with TuMV abundance. Under cold conditions, these correlations may have been weakened by a narrow range of variation in virus abundance due to slow virus accumulation. The detection of *AhgAGO2* as an upregulated DEG and the positive correlation of *AhgRDR6* with virus abundance indicated that RNA silencing was induced, at least at the gene expression level, even under cold conditions.

SA response genes were upregulated in the early stages of viral propagation, whereas RNA silencing genes were upregulated even after viral saturation. The temporal differences in defense activation found in this study are consistent with those reported from infected plants in a natural population of *A. halleri*, in which SA response genes were upregulated in early spring at earlier stages of TuMV increase (logarithmic amplification phase) and RNA silencing genes were upregulated in early autumn when TuMV abundance was saturated (Honjo et al. 2020).

Viral infections may disrupt the defense against other types of pathogens. We observed that the “defense response to bacterium” and “response to bacterium” GOs were enriched in the downregulated DEGs at 28 dpi in the warm. This may indicate that the defense against bacteria is attenuated by viral infection after a long period under warm conditions. Although infection with one virus species has been reported to promote or prevent infection, virus–bacterial interactions have rarely been studied (Lamichhane and Venturi 2015). Whether the downregulation of bacterial defense genes by TuMV promotes bacterial infection should be investigated in future studies.

Some suberin biosynthesis genes were consistently upregulated in TuMV-inoculated leaves at all time points under cold conditions (constant DEGs in Fig. 6h and i). Suberin polymer synthesis is induced in response to abiotic and biotic stresses and serves as a barrier for transporting substances between cells (Batsale et al. 2021, Kashyap et al. 2021). Further studies are required to understand the role of suberin in the movement of viruses between cells.

## Conclusions

In this study, we showed that virus–plant interactions are highly temperature-dependent. We successfully identified TuMV-responsive genes under cold conditions. TuMV used in this study was derived from a natural population of *A. halleri*. In this virus–host system, TuMV infection persists in its natural habitat for many years without causing severe damage (Honjo et al. 2020). Our results support the previous observation that the virus–host interaction fluctuates seasonally at the symptomatic and transcriptomic levels during the season and that cold in winter is the key to the persistence of infection, allowing the host to control virus accumulation and recover from symptoms (Honjo et al. 2020). Sixty-seven of the 117 DEGs reported between TuMV-infected and uninfected plants in the natural population (Honjo et al. 2020) were detected as DEGs in the present study, indicating that our experiment partially reproduced what has been observed in the natural environment. Nearly half of these genes were detected as DEGs multiple times in our experiments, including constantly upregulated DEGs (e.g. *AhgISTL6* and *AhgPP2-B1*) and DEGs with dose-dependency on viral accumulation levels, including RNA silencing genes (e.g. *AhgAGO1, AhgAGO2*, and *AhgRDR6*). To understand how plants and viruses interact and adapt to temperature fluctuations in natural environments, it is necessary to elucidate the molecular mechanisms underlying plant responses and viral accumulation over a wide range of temperatures in future studies.

## Materials and Methods

### Study systems

We obtained materials from a persistent natural system between plant and virus, *A. halleri* [*Arabidopsis halleri* (L.) O’Kane and Al-Shehbaz subsp. *gemmifera* (Matsum.) O’Kane and Al-Shehbaz] and TuMV (Honjo et al. 2020). TuMV is a Potyvirus with a positive-stranded RNA genome. The host, *A. halleri*, is a perennial herb that bears leaves throughout the year. Rosettes grow continuously during summer and autumn and then overwinter. The flowering season extends from April to May, and plants reproduce sexually by producing seeds and clonally by forming vegetative rosettes (Honjo et al. 2019). All plant and virus materials in this study were obtained from a natural population of *A. halleri* at the Omoide gawa study site, Naka-ku, Taka-cho, Hyogo prefecture, Japan (35° 06ʹ N, 134° 55ʹ E, 190–230 m in altitude) (Kudoh et al. 2018). The TuMV infection rate was reported to be 25%–57% in our previous research conducted at the study site and in a neighboring *A. halleri* population (Kamitani et al. 2016, Honjo et al. 2020).

### Growth experiment

Field-collected TuMV-free plants were used to propagate the uninfected clonal rosettes. Three uninfected (genetically different) plants (genotypes) were collected on 22 December 2015. They were incubated in a growth chamber at 20°C/15°C, day/night at 12-h day length for 20 days, and then the temperature condition was changed to 25°C/15°C, day/night at 12-h day length to promote bolting and flowering stems. They form clonal rosettes on the lateral meristems along elongated flowering stalks. After the clonal rosettes produced roots, the rosettes were separated by cutting the flowering stalks and were transplanted to new pots with soil [a 1:1 mixture of pumice (Kanuma pumice, fine-grained) and culture soil (N, P, K = 320, 210, 300 mg/l, Takii, Japan)]. These plants were grown in a growth chamber (25°C/15°C, day/night at 12-h day length) for ∼2 months until they were used for the experiment. Finally, 36 clonal rosettes from each mother plant were selected for the experiment for a total of 108 plants. At the beginning of the experiment, the average number of leaves per plant was 15.9 ± 4.4 (SD). Half of them were used for the TuMV-inoculated treatment (virus+), and the other half were used for the mock-inoculated control (virus−).

To prepare the viral inoculum, we used a TuMV-infected plant collected in September 2015 and maintained it in our laboratory. Infected leaf tissue was harvested and immediately homogenized with a cylindrical metal bead in 500 μl of PBS buffer using a mechanical homogenizer (Multi-Beads Shocker, Yasui Kikai, Japan). A constant volume (50 μl) of viral inoculum was rub-inoculated with Carborundum 600 mesh (SiC particles, <600 μm in diameters, Nacalai, Japan) onto two leaves for each plant, i.e. the ninth and 10th leaves (>2 mm in length) from the top. For mock-inoculated plants, PBS without TuMV was inoculated using the same procedure.

In the experiment, we prepared two temperature conditions, i.e. cold (10°C/5°C, day/night at 12-h day length) and warm (25°C/20°C, day/night at 12-h day length), using a biological incubator with six separate chambers (LH-80WLED-6CT, N.K. System, Japan). Three chambers were used at each temperature. For each set of virus+ and virus− treatments, 54 plants (18 clonal replicates/mother plant × 3 original mother plants) were divided into six groups, each consisting of nine plants (3 replicates × 3 origins). Three of the six groups were transferred to the cold-condition chambers, and the rest were transferred to the warm-condition chambers. The average PAR at the pot surface was 59.1 μM/S/m^2^, and the average ratio of red to far-red was 7.65.

For each of virus+ and virus− (mock) treatments, three plants (1 replicate × 3 genotypes) were harvested from each chamber (a total of 3 plants × 3 chambers × 2 temperature conditions = 18 plants were harvested) at 8, 15, and 28 dpi (day of postinfection). For each harvested plant, two inoculated leaves (pooled); the first, fourth, and seventh extant leaves from the inoculated leaves; and a new leaf (second-youngest leaf) were sampled separately to quantify TuMV. Extant and new leaves represent those present at inoculation and those newly expanded after inoculation, respectively. The SPAD values of the largest leaves, from the first to third leaves, were measured at three locations within the leaves. The fifth and sixth upper leaves of the inoculated plants were harvested and pooled into one RNA-seq sample for each harvested plant. Each sample was placed in a 2-ml screw-capped microtube and immediately frozen in liquid nitrogen. The samples were stored at −80°C until analysis. TuMV levels were quantified using RT-qPCR for all virus+ samples. RNA-seq analyses were performed on samples at 28 dpi in the cold treatment and 8, 15 and 28 dpi in the warm treatment. For the samples collected at 8 and 15 dpi in the cold treatment, RNA-seq was not performed because RT-qPCR analysis indicated that TuMV had not yet significantly increased in the upper leaves of the virus + plants. Three plants (each from 8 and 28 dpi in warm and at 28 dpi under cold conditions) were excluded from the analyses because of the failure of TuMV inoculation, as judged by the RT-qPCR results in inoculated leaves.

### Shoot dry weight

Shoot dry weight was measured for all plants on all sampling days. The remaining shoots after leaf sampling were dried in an oven (60°C, >1 week). After cooling to room temperature, the shoots were weighed. In addition, leaf length was measured in all sampled leaves using RT-qPCR and RNA-seq to estimate dry weight. The dry weight of the harvested leaves was calculated using a calibration curve for leaf length generated in advance.

### RT-qPCR

Total RNA was extracted with a Maxwell® 16 instrument using the Maxwell® 16 LEV Plant RNA Kit according to the manufacturer’s instructions (Promega, Wisconsin, USA). Leaves were completely crushed with cylindrical metal beads using a multibead shocker (Yasui Kikai, Japan). The amount of RNA was measured using a Quantus Fluorometer (Promega), and the quality was assessed using a Bioanalyzer (Agilent Technologies, CA, USA).

Using the extracted RNA, TuMV levels in the leaves were quantified by RT-qPCR following a previously described procedure (Honjo et al. 2020). Briefly, 200 ng of RNA was reverse-transcribed (High-Capacity cDNA Reverse Transcription Kit, Life Technologies) using oligo dT primers according to the manufacturer’s instructions. qPCR was performed with 1 µl of a 20-µl cDNA sample using QuantStudio 7 (Applied Biosystems). In all qPCR trials, relative amounts of TuMV RNA were calculated against a dilution series of pre-prepared standard cDNAs. The following primers were used: for TuMV detection, TuMVCP-F (5ʹ-TGGCTGATTACGAACTGACG-3ʹ, Honjo et al. 2020) and CP-R (5ʹ-CTGCCTAAATGTGGGTTTGG-3ʹ, Wei et al. 2013), and a reference gene AhgPP2AA3-F (5ʹ-GTATGCACATGTTTTGCTTCCAC-3ʹ) and AhgPP2AA3-R (5ʹ-CAACCAAGTCATTCTCCCTCATC-3ʹ) (Nishio et al. 2016). Each 10-µl PCR reaction mixture contained 1 µl cDNA, 200 nM primers, and Fast SYBR Green Master Mix (Applied Biosystems). The PCR conditions were 95°C for 20 s, followed by 40 cycles of 95°C for 1 s and 60°C for 20 s. Two replicates were performed for each sample and the standard cDNA.

### RNA-seq analysis

To evaluate the effects of TuMV infection on the host transcriptome, mRNA purification and library preparation for RNA-seq were performed according to BrAD-seq (Townsley et al. 2015) with modifications. The total RNA (500 ng/sample) was used for library preparation. Concentrated lysis/binding buffer (LBB) (×1.75) was mixed with 500 μg/30 μl of total RNA to give a final concentration of 100 mM Tris-HCl, 1 M LiCl,10 mM EDTA, 1% SDS, 5 mM DTT, 1.5% Antifoam A, and 1 mM 2-mercaptoethanol and then incubated for 5 min at room temperature. The RNA in LBB was mixed with 12.5 μM biotin-20nt-20T oligo and Streptavidin beads (Thermo Fisher Scientific, MA, USA) and then mRNA (containing TuMV genome) with poly-A tail bound to Streptavidin beads was purified by washing once with cold washing buffer A (WBA), washing buffer B (WBB), and low-salt buffer (LSB). After quantification of the mRNA using Quantus Fluorometer (Promega), strand-specific 3ʹ Digital Gene Expression (DGE) type library was prepared. Each sample was indexed with one of the 96 unique tags, and five libraries of 36 samples/libraries were prepared by mixing multiple indexed samples (total *N* = 180). Library quality was checked using a bioanalyzer. Thirty-six tagged samples were mixed with the library and sequenced. Single-read 50 base pairs and index sequencing were performed on a HiSeq 2500 (Illumina) using the TruSeq v3 platform.

Preprocessing and quality filtering were performed using Trimmomatic-v0.36 (Bolger et al. 2014). Reference sequences (v4.0) containing the nuclear and chloroplast transcript sequences of *A. halleri* (Asaf et al. 2017, Briskine et al. 2017), rRNA sequences, 8109 viral sequences (National Center for Biotechnology Information [NCBI]), and the External RNA Controls Consortium (ERCC) spike in the control (Life Technologies) were used. Transcripts of *A. halleri* (32,551 genes) (Briskine et al. 2017) were annotated using the BLAST best hit against Araport11 (Cheng et al. 2017), and gene IDs providing g and the following number (gXXXXX) were used (Briskine et al. 2017, DryAd doi:10.5061/dryad.gn4hh). The preprocessed sequences were mapped to the reference and quantified using RNA-Seq by Expectation-Maximization (RSEM) v1.2.31 (Li and Dewey 2011) and Bowtie2 v.2.2.9 (Langmead and Salzberg 2012). We used the Bowtie2 setting, designated as the default in RSEM, for the mapping accuracy parameters (bowtie2 -q --phred33 --sensitive --dpad 0 --gbar 99999999 --mp 1,1 --np 1--score-min L,0,-0.1 -p 6 -k 200 -x). The three genotypes used in this experiment likely exhibited allelic variations in the RNA-seq reads. The presence of such variations can be handled by the Bowtie2 algorithm, which primarily maps reads to the 100% matched position in the reference genome; otherwise, the best position is sought step-by-step, allowing for sequence mismatch. The output of the RSEM was analyzed using R 3.1.1 software (R Development Core Team, 2011). Host gene expression and TuMV levels were calculated as reads per million (RPM) based on the expected RSEM counts (Kamitani et al. 2016). The total reads derived from the host genes, excluding rRNA, were used as the denominator. Further host transcriptome analysis was performed using the expressed genes with an average raw count of ≥10 reads across all samples and four or more samples with a raw count of ≥1 read.

To evaluate the effects of TuMV and temperature on the host transcriptome, PERMANOVA was performed on all dpi samples of TuMV- and mock-inoculated leaves, and on each dpi sample separately, using the adonis2 function in the vegan package for R4.3.2 software. To compare and illustrate the host transcriptome pattern, PCA was performed on all dpi samples of TuMV- and mock-inoculated leaves under warm and cold conditions using the prcomp function with normalization in R4.0.4. For each gene, the log_2_ value of the RPM was used for the PERMANOVA and PCA.

DEGs were identified by comparing mock- and TuMV-inoculated plants using the EdgeR (Robinson et al. 2010). This was performed for 10 dpi–position–temperature combinations of inoculated leaves and systemic leaves at 8, 15, and 28 dpi under warm and cold conditions, except for systemic leaves at 8 and 15 dpi under cold conditions. Corrections for multiple tests were performed by setting a (false discovery rate) (Benjamini and Hochberg 1995). GO analyses were applied to DEGs using the GO.db package from the Bioconductor project for the R4.0.4 software, following the method described in our previous study (Nagano et al. 2019), and we used an updated GO list for the *A. halleri* transcriptome by applying GO terms for *A. thaliana* (TAIR10, http://www.arabidopsis.org/) to sequence homologs in *A. halleri*. We performed *K*-means clustering for the top 2000 genes that showed high variation across treatment combinations using R. We used SD as a criterion to select 2000 genes, and *k* was selected as six based on the elbow method. We performed a weighted gene coexpression network analysis (WGCNA) for all samples, including TuMV- and mock-inoculated leaves and systemic leaves, under all temperature and dpi combinations, using the R package for WGCNA (Langfelder and Horvath 2008) with the default filtration process. We used all genes expressed in the WGCNA. Gene networks were visualized using Cytoscape v. 3.10.2 (Shannon et al. 2003).

## Supplementary Material

pcaf010_Supp

## Data Availability

The data underlying this article are available in the DDBJ at https://www.ddbj.nig.ac.jp/dra/ and can be accessed by the BioProject number PRJDB19067 (PSUB024253).

## References

[R1] Aikawa S., Kobayashi M.J., Satake A., Shimizu K.K. and Kudoh H. (2010) Robust control of the seasonal expression of the *Arabidopsis FLC* gene in a fluctuating environment. *Proc. Natl. Acad. Sci. U.S.A*. 107: 11632–11637. 20534541 10.1073/pnas.0914293107 PMC2895080

[R2] Aparicio F., Thomas C.L., Lederer C., Niu Y., Wang D. and Maule A.J. (2005) Virus induction of heat shock protein 70 reflects a general response to protein accumulation in the plant cytosol. *Plant Physiol*. 138: 529–536. 15805473 10.1104/pp.104.058958 PMC1104204

[R3] Asaf S., Khan A., Khan L., A. M., Waqas M., Kang S.M., et al. (2017) Chloroplast genomes of *Arabidopsis halleri* ssp. *gemmifera* and *Arabidopsis lyrata* ssp. *petraea*: structures and comparative analysis. *Sci. Rep*. 7: 7556. 10.1038/s41598-017-07891-5 PMC5548756 28790364

[R4] Bartling D., Seedorf M., Mithöfer A. and Weiler E.W. (1992) Cloning and expression of an Arabidopsis nitrilase which can convert indole-3-acetonitrile to the plant hormone, indole-3-acetic acid. *Eur. J. Biochem*. 205: 417–424. 1555601 10.1111/j.1432-1033.1992.tb16795.x

[R5] Batsale M., Bahammou D., Fouillen L., Mongrand S., Joubès J. and Domergue F. (2021) Biosynthesis and functions of very-long-chain fatty acids in the responses of plants to abiotic and biotic stresses. *Cells* 10: 1284. 10.3390/cells10061284 PMC8224384 34064239

[R6] Benjamini Y. and Hochberg Y. (1995) Controlling the false discovery rate: a practical and powerful approach to multiple testing. *J.R. Stat. Soc. B* 57: 289–300.

[R7] Bolger A.M., Lohse M. and Usadel B. (2014) Trimmomatic: a flexible trimmer for Illumina sequence data. *Bioinformatics* 30: 2114–2120. 24695404 10.1093/bioinformatics/btu170 PMC4103590

[R8] Briskine R.V., Paape T., Shimizu-Inatsugi R., Nishiyama T., Akama S., Sese J., et al. (2017) Genome assembly and annotation of *Arabidopsis halleri*, a model for heavy metal hyperaccumulation and evolutionary ecology. *Mol. Ecol. Resour*. 17: 1025–1036. 27671113 10.1111/1755-0998.12604

[R9] Chen H., Lai Z., Shi J., Xiao Y., Chen Z. and Xu X. (2010) Roles of arabidopsis WRKY18, WRKY40 and WRKY60 transcription factors in plant responses to abscisic acid and abiotic stress. *BMC Plant Biol*. 10: 281. 10.1186/1471-2229-10-281 PMC3023790 21167067

[R10] Cheng C.Y., Krishnakumar V., Chan A.P., Thibaud-Nissen F., Schobel S. and Town C.D. (2017) Araport11: a complete reannotation of the *Arabidopsis thaliana* reference genome. *Plant J*. 89: 789–804. 27862469 10.1111/tpj.13415

[R11] Cheng G., Yang Z., Zhang H., Zhang J. and Xu J. (2019) Remorin interacting with pCaP1 impairs Turnip mosaic virus intercellular movement but is antagonised by VPg. *New Phytol*. 225: 2122–2139. 31657467 10.1111/nph.16285

[R12] Chung B.N., Choi K.S., Ahn J.J., Joa J.H., Do K.S. and Park K.-S. (2015) Effects of temperature on systemic infection and symptom expression of turnip mosaic virus in Chinese cabbage (*Brassica campestris*). *Plant Pathol. J*. 31: 363–370. 26673094 10.5423/PPJ.NT.06.2015.0107 PMC4677745

[R13] Dufresne P.J., Thivierge K., Cotton S., Beauchemin C., Ide C., Ubalijoro E., et al. (2008) Heat shock 70 protein interaction with Turnip mosaic virus RNA-dependent RNA polymerase within virus-induced membrane vesicles. *Virology* 374: 217–227. 18222516 10.1016/j.virol.2007.12.014

[R14] Elsharkawy M.M., Elsawy M.M. and Ismail I.A. (2022) Mechanism of resistance to Cucumber mosaic virus elicited by inoculation with *Bacillus subtilis* subsp. *subtilis*. *Pest Manag. Sci*. 78: 86–94. 34437749 10.1002/ps.6610

[R15] Farrona S., Hurtado L. and Reyes J.C. (2007) A nucleosome interaction module is required for normal function of *Arabidopsis thaliana* BRAHMA. *J. Mol. Biol*. 373: 240–250. 17825834 10.1016/j.jmb.2007.07.012

[R16] Harrison B.D. (1956) Studies on the effect of temperature on virus multiplication in inoculated leaves. *Ann. Appl. Biol*. 44: 215–226.

[R17] Honjo M.N., Emura N., Kawagoe T., Sugisaka J., Kamitani M., Nagano A.J., et al. (2020) Seasonality of interactions between a plant virus and its host during persistent infection in a natural environment. *ISME J*. 14: 506–518. 31664159 10.1038/s41396-019-0519-4 PMC6976672

[R18] Honjo M.N., Kudoh H. and Picó X. (2019) *Arabidopsis halleri*: a perennial model system for studying population differentiation and local adaptation. *AoB PLANTS* 11: lz076. 10.1093/aobpla/plz076 PMC6899346 31832127

[R19] Hull R. (2014) *Plant Virology*, 5th edn. London, UK: Academic Press.

[R20] Kamitani M., Nagano A.J., Honjo M.N., Kudoh H. and Kümmerli R. (2016) RNA-Seq reveals virus–virus and virus–plant interactions in nature. *FEMS Microbiol. Ecol*. 92: fiw176. 10.1093/femsec/fiw176 PMC5854034 27549115

[R21] Kashyap A., Planas-Marquès M., Capellades M., Valls M., Coll N.S. and Ort D. (2021) Blocking intruders: inducible physico-chemical barriers against plant vascular wilt pathogens. *J. Exp. Bot*. 72: 184–198. 32976552 10.1093/jxb/eraa444 PMC7853604

[R22] Kubeš M., Yang H., Richter G.L., Cheng Y., Młodzińska E., Wang. X., et al. (2012) The *Arabidopsis* concentration-dependent influx/efflux transporter ABCB4 regulates cellular auxin levels in the root epidermis. *Plant J*. 69: 640–654. 21992190 10.1111/j.1365-313X.2011.04818.x

[R23] Kudoh H., Honjo M.N., Nishio H. and Sugisaka J. (2018) The long-term “in natura” study sites of *Arabidopsis halleri* for plant transcription and epigenetic modification analyses in natural environments. *Methods Mol. Biol*. 1830: 41–57. 30043363 10.1007/978-1-4939-8657-6_3

[R24] Lamichhane J.R. and Venturi V. (2015) Synergisms between microbial pathogens in plant disease complexes: a growing trend. *Front. Plant Sci*. 6: 385. 10.3389/fpls.2015.00385 PMC4445244 26074945

[R25] Langfelder P. and Horvath S. (2008) WGCNA: an R package for weighted correlation network analysis. *BMC Bioinf*. 9: 559. 10.1186/1471-2105-9-559 PMC2631488 19114008

[R26] Langmead B. and Salzberg S.L. (2012) Fast gapped-read alignment with Bowtie 2. *Nat. Methods* 9: 357–359. 22388286 10.1038/nmeth.1923 PMC3322381

[R27] Lebeurier G. and Hirth L. (1966) Effect of elevated temperatures on the development of two strains of tobacco mosaic virus. *Virology* 29: 385–395. 5922452 10.1016/0042-6822(66)90214-5

[R28] Lee S.B. and Suh M.-C. (2018) Disruption of glycosylphosphatidylinositol-anchored lipid transfer protein 15 affects seed coat permeability in Arabidopsis. *Plant J*. 96: 1206–1217. 30242928 10.1111/tpj.14101

[R29] Lehmann T., Janowitz T., Sánchez-Parra B., Alonso M.P., Trompetter I., Piotrowski M., et al. (2017) Arabidopsis NITRILASE 1 contributes to the regulation of root growth and development through modulation of auxin biosynthesis in seedlings. *Front. Plant Sci*. 8: 36. 10.3389/fpls.2017.00036 PMC5258727 28174581

[R30] Li B. and Dewey C.N. (2011) RSEM: accurate transcript quantification from RNA-Seq data with or without a reference genome. *BMC Bioinf*. 12: 323. 10.1186/1471-2105-12-323 PMC3163565 21816040

[R31] Li K., Wu G., Li M., Ma M., Du J., Sun M., et al. (2018) Transcriptome analysis of *Nicotiana benthamiana* infected by tobacco curly shoot virus. *Virol. J.* 15: 138. 10.1186/s12985-018-1044-1 PMC6122796 30176884

[R32] Liu D., Zhao Q., Cheng Y., Li D., Jiang C., Cheng L., et al. (2019) Transcriptome analysis of two cultivars of tobacco in response to Cucumber mosaic virus infection. *Sci. Rep*. 9: 3124. 10.1038/s41598-019-39734-w PMC6395745 30816259

[R33] McLean B.G., Waigmann E., Zambryski P. and Zambryski P. (1993) Cell-to-cell movement of plant viruses. *Trends Microbiol*. 1: 105–109. 8143117 10.1016/0966-842x(93)90116-9

[R34] Nagano A.J., Kawagoe T., Sugisaka J., Honjo M.N., Iwayama K. and Kudoh H. (2019) Annual transcriptome dynamics in natural environments reveals plant seasonal adaptation. *Nat. Plants* 5: 74–83. 30617252 10.1038/s41477-018-0338-z

[R35] Nagy P.D., Wang R.Y., Pogany J., Hafren A. and Makinen K. (2011) Emerging picture of host chaperone and cyclophilin roles in RNA virus replication. *Virology* 411: 374–382. 21295323 10.1016/j.virol.2010.12.061

[R36] Nie X., Liang Z., Nie B., Murphy A. and Singh M. (2015) Studies on varietal response to different strains of Potato virus Y (PVY) reveal hypersensitive resistance in exploits to PVYO and extreme resistance in F87084 to all tested strains. *Am. J. Potato Res*. 92: 23–31.

[R37] Nishio H., Buzas D.M., Nagano A.J., Iwayama K., Ushio M. and Kudoh H. (2020) Repressive chromatin modification underpins the long-term expression trend of a perennial flowering gene in nature. *Nat. Commun*. 11: 2065. 10.1038/s41467-020-15896-4 PMC7195410 32358518

[R38] Nishio H., Buzas D.M., Nagano A.J., Suzuki Y., Sugano S., Ito M., et al. (2016) From the laboratory to the field: assaying histone methylation at *FLOWERING LOCUS C* in naturally growing *Arabidopsis halleri*. *Genes Genet. Syst*. 91: 15–26. 27150718 10.1266/ggs.15-00071

[R39] Normanly J., Grisafi P., Fink G.R. and Bartel B. (1997) Arabidopsis mutants resistant to the auxin effects of indole-3-acetonitrile are defective in the nitrilase encoded by the NIT1 gene. *Plant Cell* 9: 1781–1790. 9368415 10.1105/tpc.9.10.1781 PMC157021

[R40] Obrępalska-Stęplowska A., Renaut J., Planchon S., Przybylska A., Wieczorek P., Barylski J., et al. (2015) Effect of temperature on the pathogenesis, accumulation of viral and satellite RNAs and on plant proteome in peanut stunt virus and satellite RNA-infected plants. *Front. Plant Sci*. 6: 903. 10.3389/fpls.2015.00903 PMC4625170 26579153

[R41] Ohsato S., Miyanishi M. and Shirako Y. (2003) The optimal temperature for RNA replication in cells infected by soil-borne wheat mosaic virus is 17°C. *J. Gen. Virol*. 84: 995–1000. 12655102 10.1099/vir.0.19021-0

[R42] Padmanabhan M.S., Goregaoker S.P., Golem S., Shiferaw H. and Culver J.N. (2005) Interaction of the tobacco mosaic virus replicase protein with the Aux/IAA protein PAP1/IAA26 is associated with disease development. *J. Virol*. 79: 2549–2558. 15681455 10.1128/JVI.79.4.2549-2558.2005 PMC546588

[R43] Pierdonati E., Unterholzner S.J., Salvi E., Svolacchia N., Bertolotti G., Dello Ioio R., et al. (2019) Cytokinin-dependent control of GH3 Group II family genes in the *Arabidopsis* root. *Plants* 8: 94. 10.3390/plants8040094 PMC6524372 30965632

[R44] Qin X.F., Holuigue L., Horvath D.M. and Chua N.H. (1994) Immediate early transcription activation by salicylic acid via the cauliflower mosaic virus as-1 element. *Plant Cell* 6: 863–874. 8061520 10.1105/tpc.6.6.863 PMC160484

[R45] Qiu S., Chen X., Zhai Y., Cui W., Ai X., Rao S., et al. (2021) Downregulation of light-harvesting complex II induces ROS-mediated defense against turnip mosaic virus infection in *Nicotiana benthamiana*. *Front Microbiol*. 12: 690988. 10.3389/fmicb.2021.690988 PMC8287655 34290685

[R46] Rashid A. and Deyholos M.K. (2011) PELPK1 (At5g09530) contains a unique pentapeptide repeat and is a positive regulator of germination in *Arabidopsis thaliana*. *Plant Cell Rep*. 30: 1735–1745. 21559969 10.1007/s00299-011-1081-3

[R47] R Development Core Team 2011 R: A language and environment for statistical computing. R Foundation for Statistical Computing https://www.r-project.org/

[R48] Robinson M.D., McCarthy D.J. and Smyth G.K. (2010) edgeR: a bioconductor package for differential expression analysis of digital gene expression data. *Bioinformatics* 26: 139–140. 19910308 10.1093/bioinformatics/btp616 PMC2796818

[R49] Roossinck M.J. (2012) Plant virus metagenomics: biodiversity and ecology. *Annu. Rev. Genet*. 46: 359–369. 22934641 10.1146/annurev-genet-110711-155600

[R50] Satake A., Kawagoe T., Saburi Y., Chiba Y., Sakurai G. and Kudoh H. (2013) Forecasting flowering phenology under climate warming by modelling the regulatory dynamics of flowering-time genes. *Nat. Commun*. 4: 2303. 10.1038/ncomms3303 23941973

[R51] Seung D., Soyk S., Coiro M., Maier B.A., Eicke S. and Zeeman S.C. (2015) Protein targeting to starch is required for localising granule-bound starch synthase to starch granules and for normal amylose synthesis in *Arabidopsis*. *PLoS Biol*. 13: e1002080. 10.1371/journal.pbio.1002080 PMC4339375 25710501

[R52] Shannon P., Markiel A., Ozier O., Baliga N.S., Wang J.T., Ramage D., et al. (2003) Cytoscape: a software environment for integrated models of biomolecular interaction networks. *Genome Res*. 13: 2498–2504. 14597658 10.1101/gr.1239303 PMC403769

[R53] Suh M.C. and Go Y.S. (2014) DEWAX-mediated transcriptional repression of cuticular wax biosynthesis in *Arabidopsis thaliana*. *Plant Signal. Behav*. 9: e29463. 10.4161/psb.29463 PMC4203645 25763625

[R54] Szittya G., Silhavy D., Molnár A., Havelda Z., Lovas A., Lakatos L., et al. (2003) Low temperature inhibits RNA silencing-mediated defence by the control of siRNA generation. *EMBO J*. 22: 633–640. 12554663 10.1093/emboj/cdg74 PMC140757

[R55] Townsley B.T., Covington M.F., Ichihashi Y., Zumstein K. and Sinha N.R. (2015) BrAD-seq: Breath Adapter Directional sequencing: a streamlined, ultra-simple and fast library preparation protocol for strand specific mRNA library construction. *Front. Plant Sci*. 6: 366. 10.3389/fpls.2015.00366 PMC4441129 26052336

[R56] Wang Q., De Gernier H., Duan X.L., Xie Y.M., Geelen D., Hayashi K., et al. (2023) GH3-mediated auxin inactivation attenuates multiple stages of lateral root development. *New Phytol*. 240: 1900–1912. 37743759 10.1111/nph.19284

[R57] Waters E.R. and Vierling E. (2020) Plant small heat shock proteins – evolutionary and functional diversity. *New Phytol*. 227: 24–37. 32297991 10.1111/nph.16536

[R58] Wei T., Zhang C., Hou X., Sanfaçon H., Wang A. and Nagy P.D. (2013) The SNARE protein Syp71 is essential for turnip mosaic virus infection by mediating fusion of virus-induced vesicles with chloroplasts. *PLoS Pathog*. 9: e1003378. 10.1371/journal.ppat.1003378 PMC3656112 23696741

[R59] Whitham S.A., Quan S., Chang H.-S., Cooper B., Estes B., Zhu T., et al. (2003) Diverse RNA viruses elicit the expression of common sets of genes in susceptible *Arabidopsis thaliana* plants. *Plant J*. 33: 271–283. 12535341 10.1046/j.1365-313x.2003.01625.x

[R60] Whitham S.A., Yang C. and Goodin M.M. (2006) Global impact: elucidating plant responses to viral infection. *Mol. Plant-Microbe Interact*. 19: 1207–1215. 17073303 10.1094/MPMI-19-1207

[R61] Xu X., Chen C., Fan B. and Chen Z. (2006) Physical and functional interactions between pathogen-induced *Arabidopsis* WRKY18, WRKY40, and WRKY60 transcription factors. *Plant Cell* 18: 1310–1326. 16603654 10.1105/tpc.105.037523 PMC1456877

[R62] Yamaguchi-Shinozaki K. and Shinozaki K. (2006) Transcriptional regulatory networks in cellular responses and tolerance to dehydration and cold stresses. *Annu. Rev. Plant Biol*. 57: 781–803. 16669782 10.1146/annurev.arplant.57.032905.105444

[R63] Zhang P., Wang R., Yang X., Ju Q., Li W., Lü S., et al. (2020) The R2R3-MYB transcription factor AtMYB49 modulates salt tolerance in *Arabidopsis* by modulating the cuticle formation and antioxidant defence. *Plant Cell Environ*. 43: 1925–1943. 32406163 10.1111/pce.13784

[R64] Zhang Q., Gong M., Xu X., Li H. and Deng W. (2022) Roles of auxin in the growth, development, and stress tolerance of horticultural plants. *Cells* 11: 2761. 10.3390/cells11172761 PMC9454831 36078168

[R65] Zhao F., Yanan L., Chen L., Zhu L., Ren H. and Lin H. (2016) Temperature dependent defence of *Nicotiana tabacum* against cucumber mosaic virus and recovery occurs with the formation of dark Green Islands. *J. Plant Biol*. 59: 293–301.

[R66] Zheng S., Hu H., Ren H., Yang Z., Qiu Q., Qi W., et al. (2019) The Arabidopsis H3K27me3 demethylase JUMONJI 13 is a temperature and photoperiod dependent flowering repressor. *Nat. Commun*. 10: 1303. 10.1038/s41467-019-09310-x PMC6428840 30899015

[R67] Zhu L., Guo J., Ma Z., Wang J. and Zhou C. (2018) *Arabidopsis* transcription factor MYB102 increases plant susceptibility to aphids by substantial activation of ehylene biosynthesis. *Biomolecules* 8: 39. 10.3390/biom8020039 PMC6023100 29880735

