# Supplementary material for: Cold suppresses virus accumulation and alters the host transcriptomic response in the turnip mosaic virus–Arabidopsis halleri system
Source: Plant Cell Physiol. 2025 Jan 18;66(4):596–615. doi: 10.1093/pcp/pcaf010 (PMC12085085; doi:10.1093/pcp/pcaf010)
Supplement: pcaf010_Supp [file pcaf010_supp.zip › suppl_data/pcp-2024-e-00092-File003.pdf]

**Supplementary Figure legends**

Figure S1. | Principal component analysis (PCA) on the whole transcriptome for all samples of inoculated leaves, representing overall host transcriptomic responses for TuMV- and mock-inoculated plants at 8, 15 and 28 dpi under the cold (10˚C/5˚C, day/night temperatures) and warm (25˚C/20˚C) conditions. PCA plots for PC1 and PC2 (**A, B**). All sample points are shown in **A**, and the distribution of sample groups (TuMV- and mock-inoculated plants in the warm and cold conditions) are shown separately for each dpi in **B**. PCA plots for PC1 and PC3 (**C, D**). All sample points are shown in **C**, and the distribution of sample groups are shown separately for each dpi in **D**. The contribution rates of the axes are given in parentheses in **A** and **C**.

Figure S2. | Analyses of differentially expressed genes (DEGs) in the mock-inoculated control to illustrate the effect of temperature (cold vs warm) in the absence of TuMV infection at different dpi and leaf position. Note that the terms, dpi and inoculated/systemic leaves, are used to represent the time and leaf position in the experiment, and TuMV-infected plants are not included in the analysis presented here. **A-D.** Number of DEGs at 8, 15 and 28 dpi for the inoculated (**A**) and systemic leaves (**B**). Upregulated and downregulated DEGs represent those with higher and lower gene expression in cold than warm conditions. For the systemic leaves in the cold, transcriptome (RNA-seq) analyses were performed only at 28 dpi. **C.** A heatmap showing the results of gene ontology (GO) enrichment analyses on DEGs between the warm and cold in the mock-inoculated control for the four position-dpi combinations. The results of 18 selected GOs in biological processes whose $-\mathrm{Log}_{10}$ (*FDR*) was higher than 10 at least at one time point are listed. The results for upregulated and downregulated DEGs are shown in red and blue colors, respectively. We performed GO analysis separately for upregulated and downregulated DEGs, and there were no cases where the same GO terms were enriched for upregulation and downregulation simultaneously. Therefore, each cell was colored when either upregulated or downregulated DEGs were enriched in the corresponding GO. Grey color represents no enrichment of the corresponding GO.

Figure S3. | A heatmap showing the results of gene ontology (GO) enrichment analyses on DEGs between TuMV- and mock-inoculated plants for the ten dpi-position-temperature combinations. We conducted GO analyses separately for upregulated and downregulated DEGs. Therefore, all significantly enriched GO

terms ($FDR < 0.05$) for upregulated and downregulated DEGs are shown separately and represented in red and blue, respectively. White color represents no enrichment of the corresponding GO ($FDR \geq 0.05$). BP: biological process, CC: cellular component, MF: molecular function.

Figure S4. |A heatmap showing expression patterns of DEGs that are classified by k-means clustering and the results of gene ontology (GO) enrichment analyses. **A.** The expression profiles of the top 2000 DEGs that showed higher variation across treatment combinations, arranged by k-means clustering ($k = 6$). **B.** Top five biological process GOs (ranked by $-\text{Log}_{10}$ ($FDR$) value) are listed for the cluster 1, 4, 5, and 6. No GO is enriched for the cluster 2 (ND). The top 8 GOs were listed for cluster 3. Dark purple, light purple, green, yellow, and blue letters represent the GO terms related to defense, abiotic stress, photosynthesis, response to light, and auxin, respectively.

Figure S5. | Gene expression patterns of DEGs between TuMV-inoculated and mock-inoculated plants belonging to the 'response to virus' GO. Heatmaps showing at which dpi-position-temperature combinations the genes were detected as DEGs ($FDR < 0.05$). Red and blue color gradients represent the fold change level for upregulated and downregulated DEGs in TuMV-inoculated plants compared to mock-inoculated plants, respectively. Grey color represents no significant enrichment.

Figure S6. | Results of the weighted gene co-expression network analysis (WGCNA) performed on all expressed genes. A heatmap of the Pearson correlation matrix between the module and selected factors, i.e., presence /absence of TuMV infection, TuMV abundance, temperature, dpi and leaf position. Red and green color gradients and values indicate the correlation coefficients ($p$-value in parentheses). Asterisks indicate the modules which showed highest positive and negative correlations with the presence/absence of TuMV infection, that is, darkolivegreen and saddlebrown, respectively.

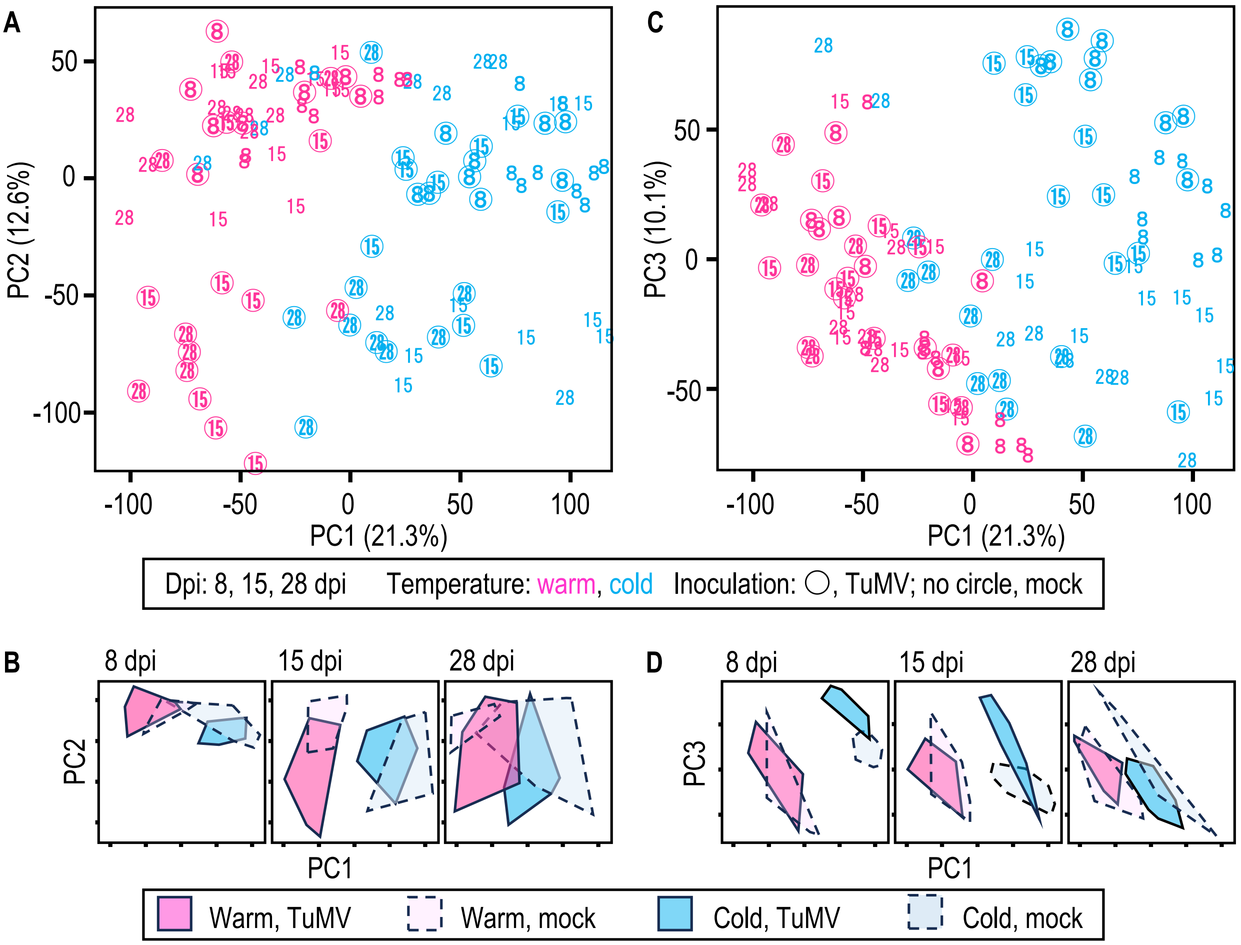


Supplementary Figure S1

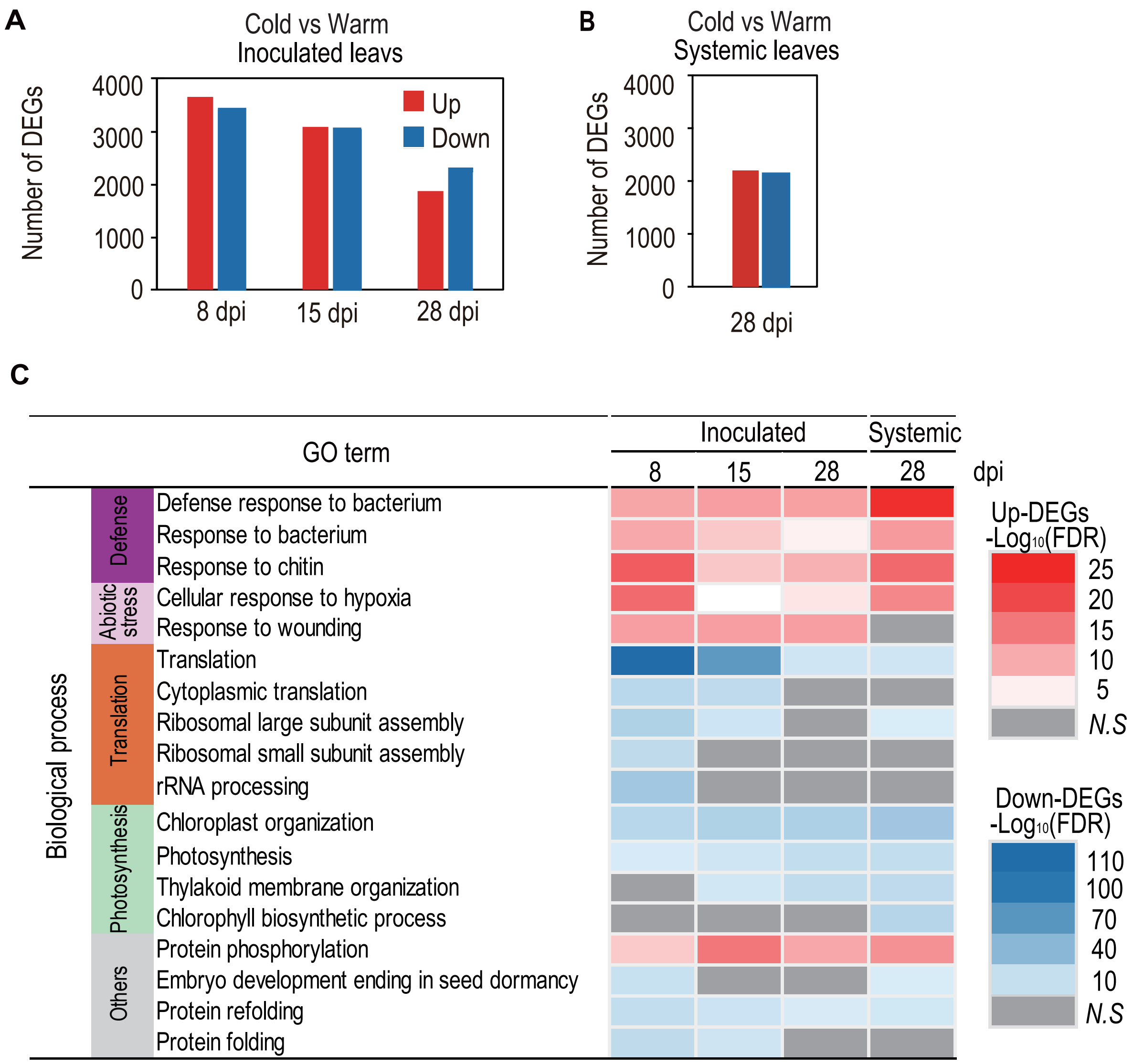


Supplementary Figure S2

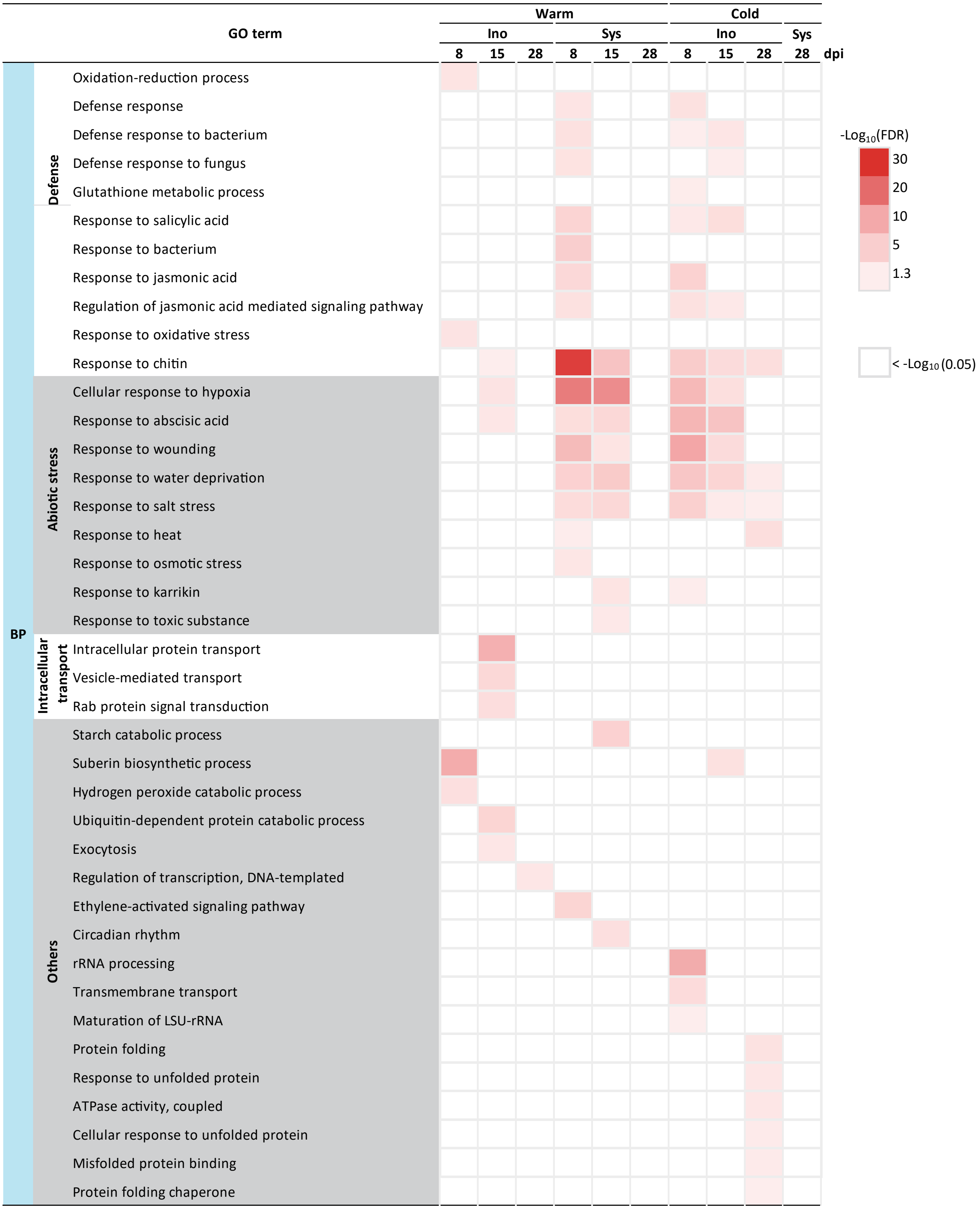


Supplementary Figure S3 (1/4)

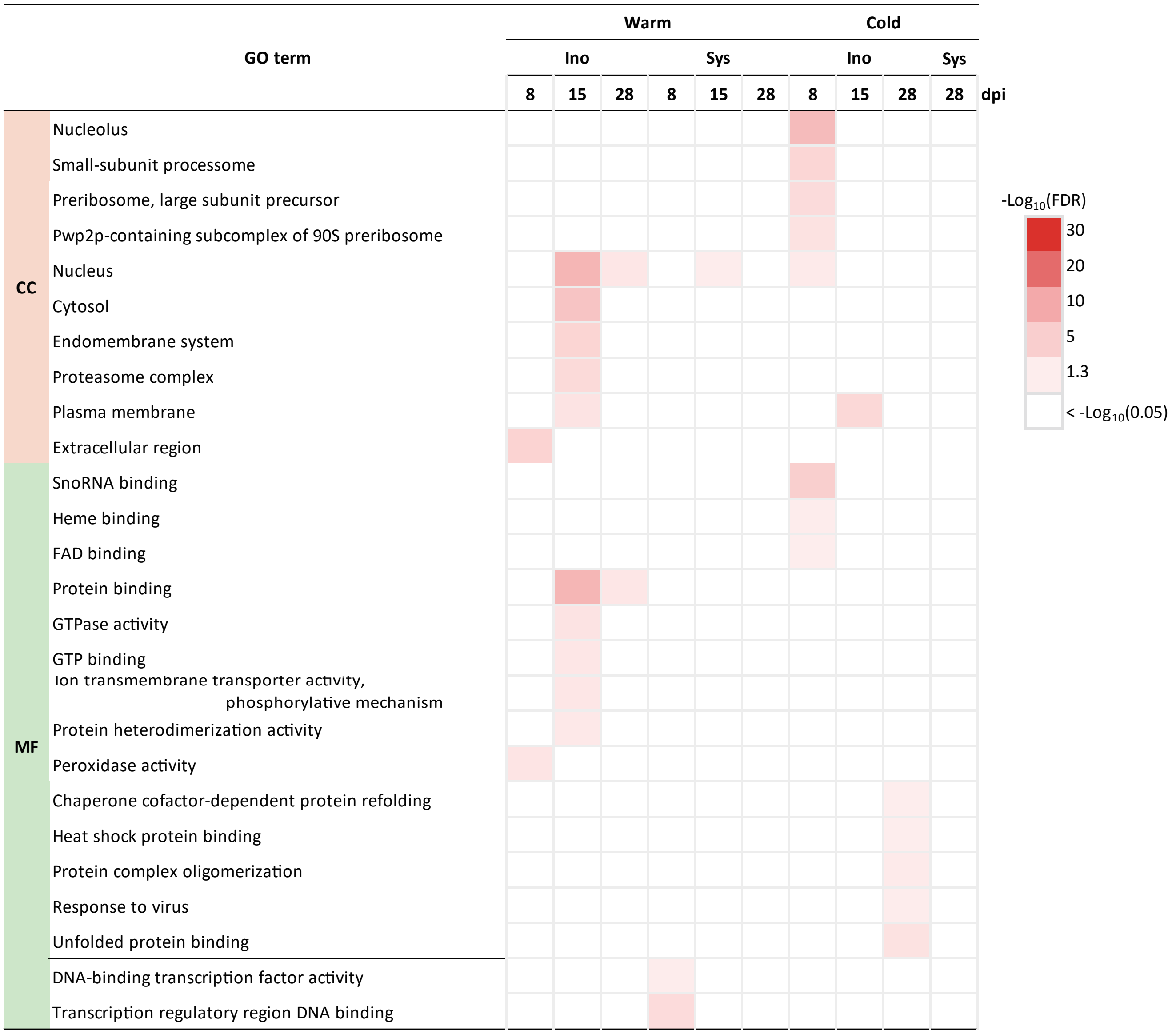


Supplementary Figure S3 (2/4)

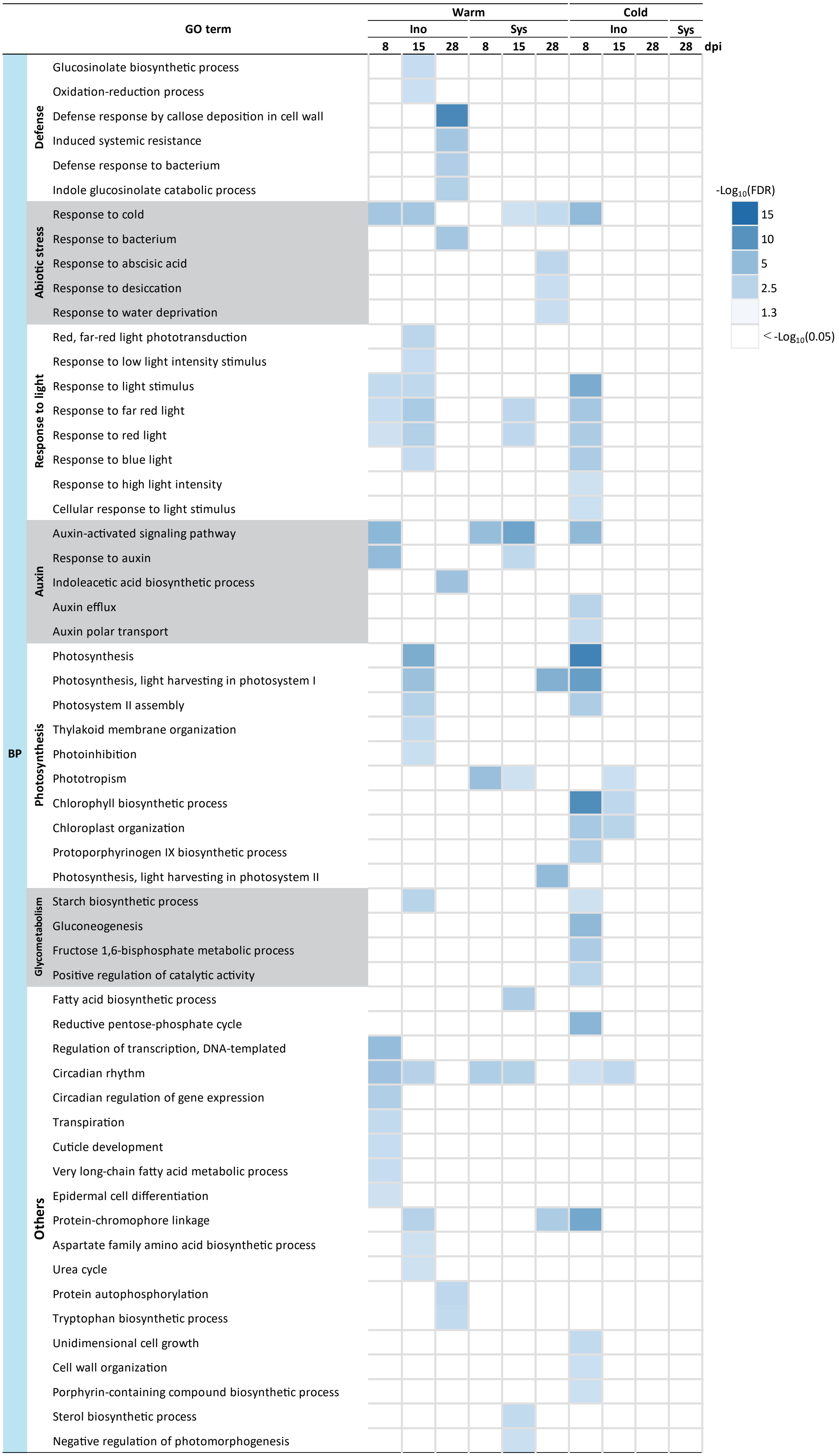


Supplementary Figure S3 (3/4)

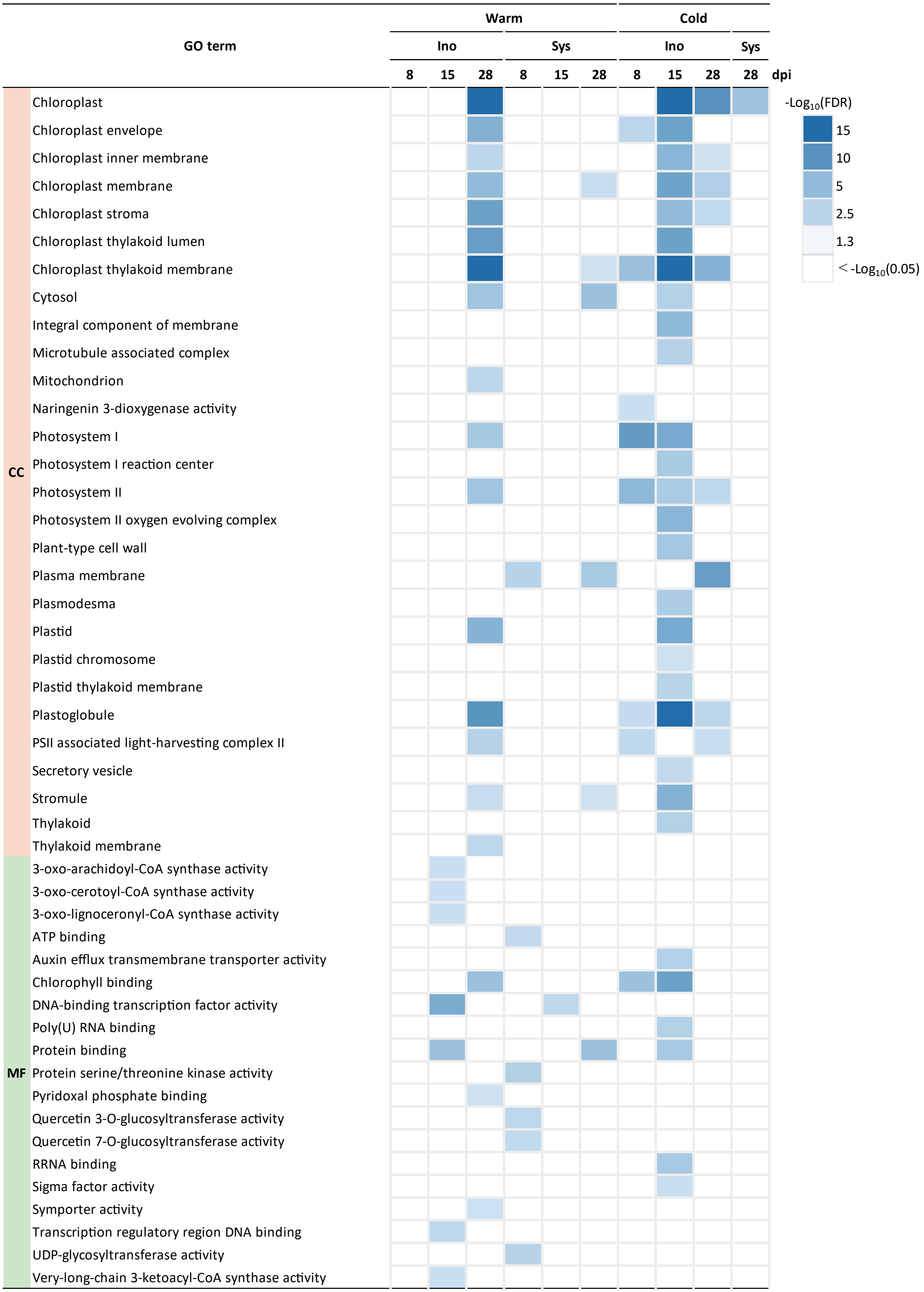


Supplementary Figure S3 (4/4)

**A**

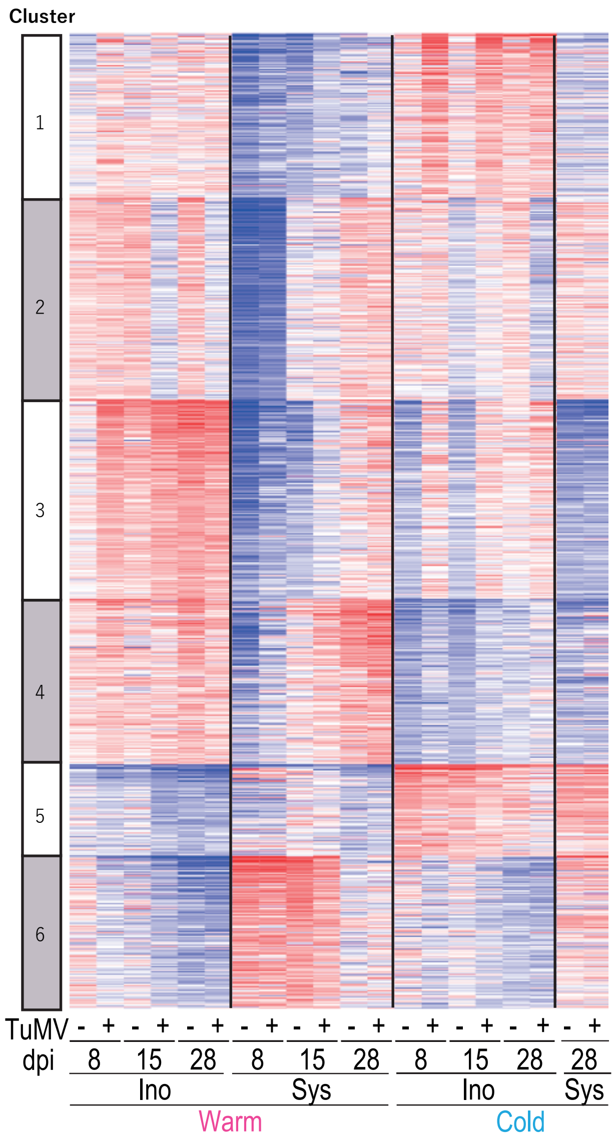


**B**

| Cluster | ID | Description | Number of DEGs[a] | -Log10 (FDR) |
|---|---|---|---|---|
| 1 | GO:0055114 | Oxidation-reduction process | 42 | 5.14 |
| | GO:0009414 | Response to water deprivation | 17 | 2.82 |
| | GO:0009409 | Response to cold | 16 | 2.08 |
| | GO:0042744 | Hydrogen peroxide catabolic process | 8 | 1.67 |
| | GO:0009651 | Response to salt stress | 16 | 1.57 |
| 2 | ND | ND | ND | ND |
| 3 | GO:0009737 | Response to abscisic acid | 31 | 8.38 |
| | GO:0010150 | Leaf senescence | 13 | 4.25 |
| | GO:0009617 | Response to bacterium | 12 | 3.72 |
| | GO:0071456 | Cellular response to hypoxia | 17 | 3.39 |
| | GO:0009414 | Response to water deprivation | 19 | 3.11 |
| | GO:0009651 | Response to salt stress | 20 | 2.56 |
| | GO:0009751 | Response to salicylic acid | 10 | 2.31 |
| | GO:0042742 | Defense response to bacterium | 16 | 2.11 |
| 4 | GO:0071456 | Cellular response to hypoxia | 31 | 17.92 |
| | GO:0010200 | Response to chitin | 26 | 17.92 |
| | GO:0042742 | Defense response to bacterium | 25 | 9.93 |
| | GO:0009611 | Response to wounding | 21 | 9.38 |
| | GO:0009617 | Response to bacterium | 15 | 8.15 |
| 5 | GO:0080167 | Response to karrikin | 9 | 2.88 |
| | GO:0010380 | Regulation of chlorophyll biosynthetic process | 4 | 2.17 |
| | GO:0010114 | Response to red light | 6 | 2.08 |
| | GO:0007623 | Circadian rhythm | 7 | 2.00 |
| | GO:0009409 | Response to cold | 11 | 1.69 |
| 6 | GO:0009768 | Photosynthesis, light harvesting in photosystem I | 9 | 8.30 |
| | GO:0009416 | Response to light stimulus | 17 | 6.73 |
| | GO:0009734 | Auxin-activated signaling pathway | 15 | 5.15 |
| | GO:0018298 | Protein-chromophore linkage | 8 | 4.97 |
| | GO:0009769 | Photosynthesis, light harvesting in photosystem II | 4 | 4.72 |

Supplementary Figure S4

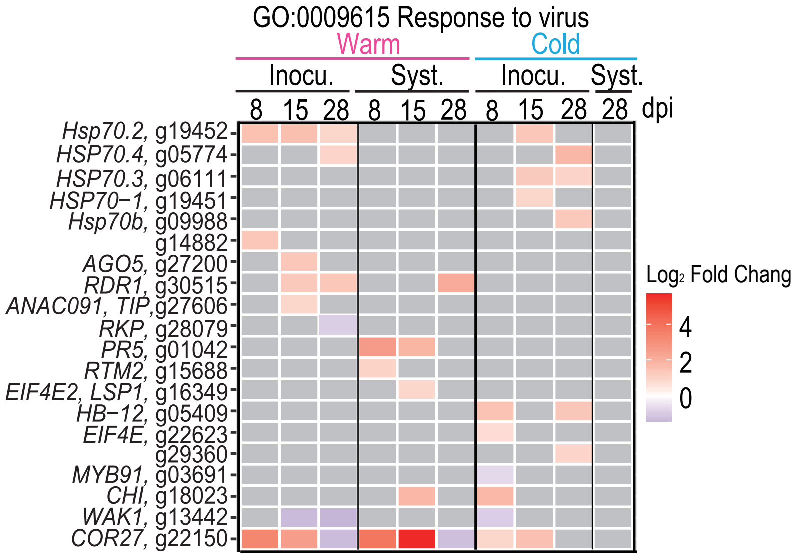


Supplementary Figure S5

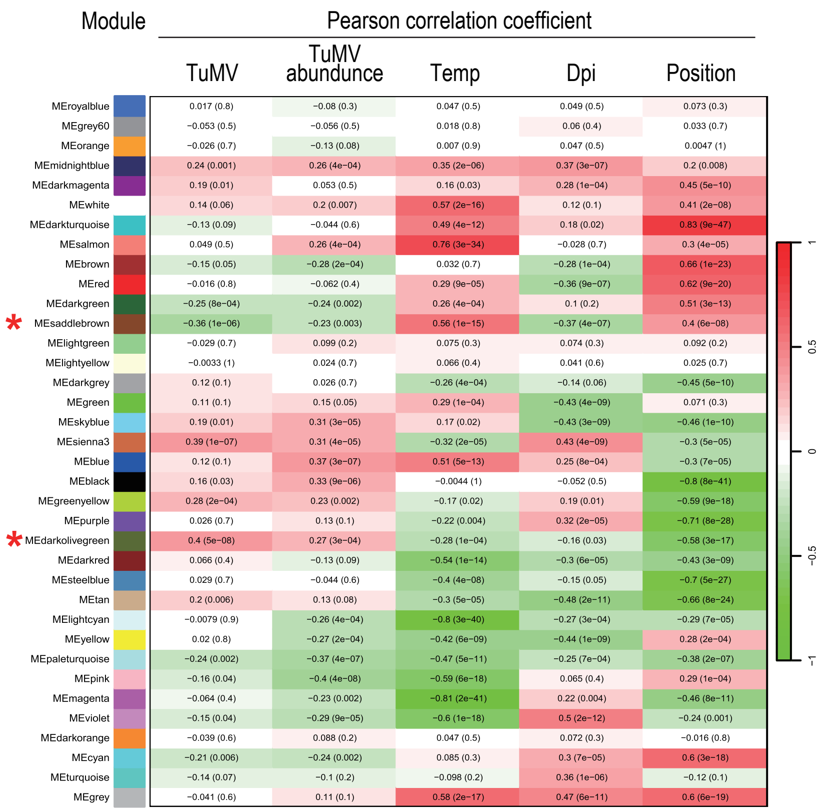


Supplementary Figure S6
